# Signatures of cell death and proliferation in perturbation transcriptomics data—from confounding factor to effective prediction

**DOI:** 10.1093/nar/gkz805

**Published:** 2019-09-25

**Authors:** Bence Szalai, Vigneshwari Subramanian, Christian H Holland, Róbert Alföldi, László G Puskás, Julio Saez-Rodriguez

**Affiliations:** 1 RWTH Aachen University, Faculty of Medicine, Joint Research Centre for Computational Biomedicine (JRC-COMBINE), 52074 Aachen, Germany; 2 Semmelweis University, Faculty of Medicine, Department of Physiology, H-1094 Budapest, Hungary; 3 Heidelberg University, Faculty of Medicine and Heidelberg University Hospital, Institute of Computational Biomedicine, Bioquant, 69120 Heidelberg, Germany; 4 Astridbio Technologies Ltd., H-6726 Szeged, Hungary

## Abstract

Transcriptional perturbation signatures are valuable data sources for functional genomics. Linking perturbation signatures to screenings opens the possibility to model cellular phenotypes from expression data and to identify efficacious drugs. We linked perturbation transcriptomics data from the LINCS-L1000 project with cell viability information upon genetic (Achilles project) and chemical (CTRP screen) perturbations yielding more than 90 000 signature–viability pairs. An integrated analysis showed that the cell viability signature is a major factor underlying perturbation signatures. The signature is linked to transcription factors regulating cell death, proliferation and division time. We used the cell viability–signature relationship to predict viability from transcriptomics signatures, and identified and validated compounds that induce cell death in tumor cell lines. We showed that cellular toxicity can lead to unexpected similarity of signatures, confounding mechanism of action discovery. Consensus compound signatures predicted cell-specific drug sensitivity, even if the signature is not measured in the same cell line, and outperformed conventional drug-specific features. Our results can help in understanding mechanisms behind cell death and removing confounding factors of transcriptomic perturbation screens. To interactively browse our results and predict cell viability in new gene expression samples, we developed CEVIChE (CEll VIability Calculator from gene Expression; https://saezlab.shinyapps.io/ceviche/).

## INTRODUCTION

Predicting cellular phenotypes (disease state, cancer drug sensitivity etc.) from high-coverage molecular (‘omics’) data is a key question of current systems biology research. Transcriptomics (microarrays or more recently RNA-Seq) is one of the key data sources for these studies due to its affordable acquisition and the well-established methodologies for analysis (1). While basal gene expression can give valuable information about cell state and function, perturbation transcriptomics signatures (i.e. measured gene expression changes after different perturbations such as drugs, gene overexpression or knockdown/knockout) provide additional possibilities to infer cellular function (2). Compounds with similar mechanism of action (MoA) tend to lead to similar transcriptomics changes, making perturbation signatures a valuable tool to identify MoA of unknown compounds (2–4). Furthermore, perturbation of different cellular pathways with pathway specific perturbagenes allows the identification of pathway-regulated genes, from which pathway activity can be effectively inferred (5,6).

Small scale studies (comprehensively collected in (7)) and the original Connectivity Map study (2) provide rich perturbation signature data. The recent release of the LINCS-L1000 dataset (4), with more than 1 000 000 signatures, increases these numbers by an order of magnitude. In the LINCS-L1000 screen, more than 20 000 different perturbagenes (compounds, shRNAs etc.) were used in dozens of different cell lines, with different concentrations and perturbation times. Importantly, these high-throughput measurements were possible based on the inexpensive L1000 methodology, that measures only ∼1000 (landmark) genes, while the rest of gene expression values were inferred. While this dataset alone opens myriads of possible applications, linking these perturbation signatures with other large scale, phenotypic studies enable the modeling of cellular function on a previously unavailable scale.

Arguably the simplest, but at the same time one of the most important cellular phenotypes is cell viability—cell death or proliferation. Analyzing cell viability data together with perturbation signatures is especially important in cancer research, as it can help to understand the mechanism of anticancer drugs, and open new therapeutic possibilities. A recent study (8) analyzed about 600 pairs of anticancer drugs and breast cancer cell lines where perturbation transcriptomics signatures (using the L1000 methodology) and cell viability were measured in parallel, leading to important information regarding cell line specific drug effects and drug synergy. There are also rich sources of other cell viability datasets available—but without the corresponding expression measurements upon perturbation. In particular, preclinical studies like GDSC (9), CTRP (10) or NCI60 (11) generated large scale cell viability datasets following drug (compound) perturbations, to identify potential drug sensitivity biomarkers. Other approaches, like project Achilles (12), used shRNA screens and created large scale gene essentiality data sources, with the aim of identifying potential new anticancer targets. These datasets partially overlap with the LINCS-L1000, allowing an integrated analysis of perturbation signatures and cell phenotype (13).

Another important aspect of cell viability and perturbation signatures is related to the fact that cell death can lead to transcriptomic changes unrelated to the perturbation, and this phenomenon can be a confounding factor to infer mechanism of action. Also one of the original LINCS-L1000 papers (14) found that some cell line and perturbation independent factor is responsible for the largest part of variability in the L1000 signatures. This factor has been hypothesized to be related to some general cell biological effects like cell viability or proliferation, but this has not been analyzed and thus remains uncertain.

In this study, we analyzed the associations between perturbation signatures and cell viability by matching (same cell line and perturbation) more than 90 000 data points between LINCS-L1000 project (perturbation signature) and the CTRP drug and Achilles shRNA screens (cell viability)—creating, to our knowledge, the largest integrative analysis of gene expression signatures and cell viability. We identified a common cell viability signature (CVS) and were able to predict cell viability effectively even across studies from different sources and types of perturbations. By analyzing the CVS, we found several transcription factors with a causal role in cell death and proliferation, and found associations between this signature and cell division time. By analyzing pairwise signature similarities, we found that the ‘cell death signature’ can lead to unexpected similarity between signatures of toxic compounds and thereby can influence the mechanism of action identification. However, using a reduced perturbation signature (removing genes showing high correlation with cell viability), we were able to reduce this effect. Our models allowed us to predict cell viability for all the compounds used in the LINCS-L1000 dataset, identifying several potential drugs with death-inducing or pro-growth properties. By using consensus compound signatures and machine learning models, we were able to predict anticancer drug sensitivity even in cell lines where the drug signature was not measured, outperforming conventional drug specific features (e.g. nominal drug targets or chemical fingerprints).

## MATERIALS AND METHODS

### Databases and data preprocessing

We used the Phase I and Phase II LINCS-L1000 perturbational profiles (4) (GSE92472 and GSE70138 respectively, downloaded from Gene Expression Omnibus (15)) in this study. Replicate-collapsed differential expression signatures (Level5 dataset) of the measured (landmark) genes were used in our analysis pipeline. For accessing L1000 signatures, we used cmapPy Python library (16). Phase I and Phase II data were merged, and signatures corresponding to the same conditions (treatment, cell line, time and concentration in case of compounds, or treatment, cell line and time in case of shRNA) were averaged using the MODZ method.

CTRPv2 cell viability dataset (10) was downloaded from CTD^2^ Data Portal (https://ocg.cancer.gov/programs/ctd2/data-portal). For further analysis the post-quality-control cell viability values were used. We matched CTRP and L1000 instances based on cell line and Broad compound IDs. For matching compound concentrations, we matched the instances between CTRP and L1000, where the concentration difference was the smallest, and the absolute log10 concentration difference was smaller than 0.2 (∼1.5-fold concentration difference). From CTRP dataset, we used percent cell viability data for creating linear models, while as summary statistics dose–response AUC was used in case of predicting NCI60 drug toxicity.

Achilles 2.4.6 and 2.19.2 datasets (12) were downloaded from Project Achilles Data Portal (https://portals.broadinstitute.org/achilles). We used the shRNA log fold change scores in our analysis (i.e. without separating on- and off-target effects of shRNAs). We matched Achilles and L1000 instances based on cell lines and shRNA treatment. As LINCS-L1000 identifies shRNAs with Construct ID and Achilles uses Barcode Sequence, we mapped these two identifiers with the help of the reference files from the Genetic Perturbation Platform (https://portals.broadinstitute.org/gpp/public/).

NCI60 drug toxicity datasets (11) (GI50, LC50 and TGI values) were downloaded from the Developmental Therapeutics Program data portal (https://dtp.cancer.gov/discovery_development/nci-60/). We restricted our analysis to those compounds that overlap between L1000 and NCI60 screens. For easier comparison, we extracted the PubChem Compound IDs using PUG REST services in R (https://pubchemdocs.ncbi.nlm.nih.gov/pug-rest-tutorial). For compounds in the NCI60 dataset, we converted Substance IDs to Compound IDs. Whereas for compounds in the L1000 dataset, we either used the Compound IDs directly (when available) or retrieved them based on the InChi keys. As the original (PubChem Compound ID based) intersection between L1000 and NCI60 datasets was relatively small (373 compounds), we matched compounds based on their name synonyms (retrieved using PubChemPy Python library) that resulted in 583 shared compounds between the two datasets.

GDSC data (expression and drug response (9)) was downloaded from https://www.cancerrxgene.org/. We used GDSC drug response summary metrics ln(IC50) and AUC in our analysis.

For accessing gCSI dataset (expression and doubling time, (17)) we used the *compareDrugScreens* R package from http://research-pub.gene.com/gCSI-cellline-data/.

### Moderated *z*-score (MODZ)

We calculated consensus signatures using a moderated *z*-score, described in the original LINCS-L1000 paper (4,14). Basically, for each set of signatures a pairwise Spearman correlation matrix was calculated. Diagonals (self correlations) were set to 0, while negative correlations were set to a small value (0.01). The weight for each signature was the sum of these correlation values row-wise (normalized so that the sum of the weights was 1). The final consensus signature was calculated as a weighted average of the signatures.

### Linear models

We used linear regression (*y* = *Xβ*) with L2 regularization (*α* = 1.0) to predict cell viability (y, n*1 column vector, where *n* is the number of samples) from perturbation gene expression signatures (X, n*g matrix, where *n* is the number of samples and *g* is the number of genes in the signatures). To evaluate prediction performance, we used a random sub-sampling validation strategy: half of a given dataset was used to train the models, while cell viability was predicted for the other half of the dataset. This process was repeated for 20 iterations and we used the Pearson correlation between the predicted and observed cell viability values as an evaluation metric. We refer ‘within dataset prediction’, when the training and the test data come from the same dataset (e.g. CTRP-L1000-24h). In contrast, when we train a model on one dataset and predict cell viability for another dataset (e.g.: CTRP-L1000-24h and Achilles-L1000-96h, respectively), we use the term ‘across dataset prediction’. In case of ‘across dataset prediction’, we trained the linear model on half of the training data but used all of the test data for evaluation. For the ‘standard’ model, feature matrix (X) was composed of vectors of cell line and perturbation IDs and a vector containing log10 drug concentration (only in case of CTRP-L1000 datasets).

### Enrichment analysis

We calculated Pearson correlation *r* values between cell viability and gene expression for each gene in the Achilles-L1000 and CTRP-L1000 datasets. We used these *r* values as input for the piano R package (18), and calculated Gene Ontology (19) and KEGG pathway (20) enrichment (fgsea method). We report FDR adjusted *P* values for the top 10 Gene Ontology terms. For transcription factor regulon enrichment, we used TF regulons from the DoRothEA framework (21,22). Normalized Enrichment Scores were calculated using the viper R package (23). For pathway activity inference, we used the PROGENy (5) method. *Z* scores for pathway activity were calculated by permuting gene labels.

### Statistical analysis of signature score associations

Cell viability signature score was calculated from (normalized) baseline expression. For associations with GDSC drug sensitivity, we fit the linear model (Sensitivity = f(CVS, Tissue, MSI), where CVS denotes the cell viability signature score of cell lines) for each drug. For comparison with random/single gene associations, we created random Achilles-based models to predict cell viability (by permuting gene or sample labels before modeling) and either calculated signature scores from these models or used expression of the L1000 genes as ‘single’ gene signature score. General level of drug sensitivity (GLDS) was calculated by fitting one linear model (Sensitivity = f(Cell, Drug), where Cell and Drug corresponds factors denoting cell and drug ID) for the whole GDSC drug sensitivity dataset, and the cell line specific coefficient of this model was used as GLDS metric for each cell line. For partial correlation calculation between GLDS (or doubling time) and signature score we fit two linear models, GLDS = f(Tissue) and CVS = f(Tissue), and calculated partial correlation as the Pearson correlation between the residuals of these two linear models.

### Average silhouette analysis

For evaluation of the different factor-based (cell line, drug, perturbation time and cell viability) clusterings of CTRP-L1000 data points in the first two principal component plane, we used average silhouette analysis with Euclidean distances. Silhouette coefficient (*b* − *a*) / max(*a*,*b*) was calculated for each datapoint, where *a* was the mean intra- and *b* was the mean nearest-cluster distance. For each clustering factor, the average of Silhouette Coefficients were calculated (scikit-learn Python library (24)). A negative average silhouette score corresponds to the absence of clustering, while a positive score means clustering of data points is based on the selected factor.

### Signature similarity analysis and MoA prediction

We analyzed mechanism of action and cell death based signature similarity using the Spearman correlation as a similarity metric. We calculated the similarity between signature pairs of the CTRP-L1000-24h dataset, where signature pairs were coming from nontoxic (cell viability > 0.8) perturbations with shared MoA, or toxic (cell viability < 0.8) perturbations with different MoA. For the MoA definition, we used compound metadata (the gene symbol of the protein target, target or activity of the compound) from the CTRP screen. For defining nontoxic and strongly toxic cell viability thresholds, we fit a Gaussian Mixed model on cell viability values (mean of nontoxic group ∼1.0, SD ∼0.1, so a threshold of 0.8 corresponds to mean − 2 * SD). For analyzing the effect of reduced signatures, we either removed random *n* genes from the perturbation signatures or the top *n* genes with the highest absolute Pearson correlation with cell viability phenotype. To prevent ‘data leakage’, the Pearson correlation between gene expression and cell viability was calculated from the Achilles-L1000-96h dataset. For residual gene expression signatures, we fit a linear model between cell viability and gene expression (G = f(CV)) for each gene, and used the residuals of these models as gene expression values.

For MoA prediction, we used the CTRP-L1000-24h and a larger part of the LINCS-L1000 dataset, where we selected compounds with known MoA based (25) on the Drug Repurposing Hub (clue.io/repurposing, May 2018 version). We calculated consensus signatures for each compound using the MODZ method, using all signatures for the given compound. For each pair of compounds, signature similarity was calculated by Spearman correlation. To analyze the prediction performance of these similarity values for mechanism of action, ROC and Precision Recall curves were used, where true positive values correspond to compounds with a shared MoA. For comparison, we calculated structure and (in case of CTRP data) sensitivity profile based similarity. Structure-based similarity was calculated by the Tanimoto similarity among chemical fingerprints (calculated by RDKit modules in Python) of compounds. For sensitivity profile similarity, we calculated Pearson correlations between the drug sensitivity AUCs for compound pairs across all cell lines. For combining signatures and chemical similarities, we simply summed the (normalized) similarity vectors.

### NCI60 validation

The NCI60 screen (11) calculated GI50 (50% growth inhibition concentration), TGI (total growth inhibition concentration) and LC50 (50% lethal concentration) as drug sensitivity metrics for the used cell line compound pairs. A sensitivity metric was only given, if the desired effect (50% growth inhibition etc.) is reached in the used concentration range, otherwise the maximal tested drug concentration is assigned. Based on this, we defined the delta concentration metric as: sensitivity metric minus the maximal tested concentration (log10 concentration values). Based on this definition of delta concentration values, delta concentrations < 0 indicate an effective drug, so this threshold was used for binarization for the ROC analysis. For the ROC analysis, we used binarized delta concentration values as true positive/negative, while the predictions of linear models were used as target scores. For each cell line—compound pair, we used the lowest predicted cell viability (when multiple signatures were available) as the target score. For the ‘ground truth’ model (predicting drug sensitivity in NCI60 from drug sensitivity in CTRP for shared cell lines and compounds), the dose–response AUC values from the CTRP screen were used.

### Cell viability assay

PC3 and VCaP cell lines were purchased from ATCC. Cytotoxicity of different test compounds were studied on both PC3 and VCaP cell lines by determining the number of viable cells based on quantitation of ATP using the CellTiter-Glo® Luminescent Cell Viability Assay (Promega, Mannheim, Germany).

Cells were seeded into 384-well plates (Corning Life Sciences, Tewksbury, MA, USA) at 1000 cells/well density in 40 μl media and incubated for 4 h at 37°C. Test compounds were dissolved in dimethyl sulfoxide (DMSO, Sigma, Budapest, Hungary) and cells were treated with an increasing concentration of drugs (1.11–90 μM). The highest applied DMSO content of the treated cells was 0.5%. After 48-h incubation at 37°C under 5% CO_2_, 40 μl CellTiter-Glo® Reagent (Promega) was added to each well and the luminescent signal was recorded by luminometer (VICTOR Multilabel Plate Reader, Perkin Elmer). Viability was calculated with relation to untreated control cells after extracting signals from blank wells containing only culture medium. IC50 values (50% inhibiting concentration) were calculated by GraphPad Prism®5 (La Jolla, CA, USA).

### Machine learning models

Transcription factor and pathway activities were calculated as cell line specific features from baseline gene expression data (9). For each *g* gene and *c* cell line, standardized gene expression was calculated as *Z*_gc_ = (*E*_gc_ − μ_g_)/σ_g_, where *E*_gc_ is gene expression, μ_g_ and σ_g_ are means and standard deviations, respectively, of a gene across cell lines. From these standardized gene expression values transcription and pathway activities were calculated using DoRothEA (21,22) and PROGENy (5,26) methods, as described previously.

Nominal target and targeted pathway features were created from manually annotated drug metadata from the GDSC portal (www.cancerrxgene.org). Extended-Connectivity Fingerprints (ECFP-like) were generated by using the RDKit fingerprint module in Python with the radius and number of bits being set to 2 and 256, respectively. For consensus signature-based features, we mapped the PubChem compound IDs of the GDSC drugs with that of the compound IDs in LINCS-L1000 dataset. For each GDSC drug, a consensus signature was calculated by using the MODZ method (using all available 24-h signatures, irrespective of cell line and concentration). To reduce the dimensionality (978) of these signature features, we performed a PCA and selected the first 40 Principal Components (explained variance: 95%).

We used Random Forest Regression models (with 50 trees) from the scikit-learn Python library (24). For each model, the specified drug features (targets, pathways, chemical fingerprints or consensus signatures) and all cell-specific features (tissue type, pathway and transcription factor activities) were concatenated to create the feature matrix. The area under the dose–response curve (AUC) was used as a drug sensitivity metric. We used a random sub-sampling strategy to train and evaluate model performance—for each run, half of the drugs were included in the training set, while the remaining half formed the test set. Three different methods were used to split the GDSC drugs into training and test sets: random splitting, splitting where for each drug in the test set there was a corresponding drug with a shared target in the training set, and splitting where all drugs targeting a given protein were either in the test or in the training set. For evaluation, the Pearson correlation was calculated for each cell line between the predicted and observed AUC values and these cell wise Pearson correlations were averaged. This random sub-sampling validation process was repeated 20 times.

### Statistical analysis

Statistical significance was calculated using the corresponding functions of SciPy library (Pearson correlation, Spearman correlation, Mann–Whitney *U* test, Fisher exact test, paired *t*-test) and ANOVA and pROC (27) from R.

## RESULTS

### Signatures of cell death in the LINCS-L1000 dataset

**Figure 1.**
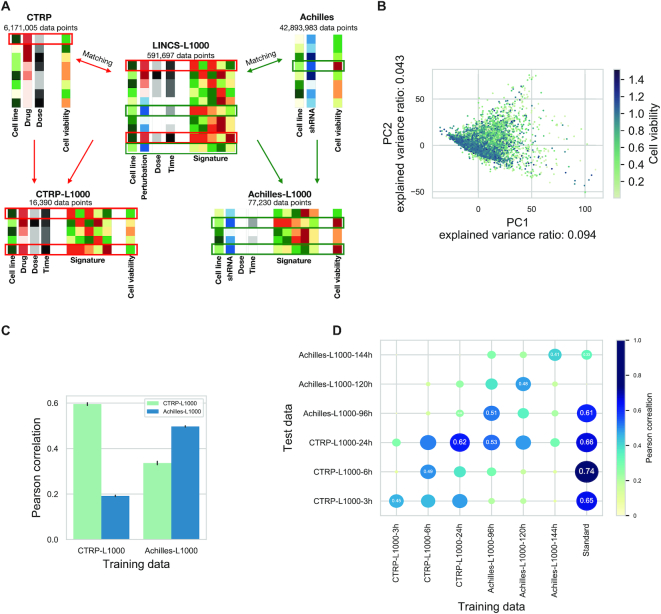
LINCS-L1000 perturbation signatures allow efficient prediction of cell viability. (**A**) Schematic representation of database matching pipeline. Perturbation signatures from LINCS-L1000 dataset were matched with cell viability data from CTRP and Achilles datasets based on metadata (cell line, perturbation and concentration). It is possible to match one CTRP/Achilles cell viability instance with more than one LINCS-L1000 signature (same cell line and perturbation but different perturbation time in LINCS-L1000). (**B**) Principal Component Analysis (PCA) of perturbation signatures from the CTRP-L1000 dataset. Each point represents a unique cell line–compound–concentration–perturbation time instance. Points are colored according to corresponding cell viability from CTRP screen (Spearman correlation between PC1 and cell viability: −0.278, *P*< 1e–300). (**C**) Prediction of cell viability using linear models. Linear models were trained on CTRP-L1000 and Achilles-L1000 datasets (*x* axis). Prediction performance was evaluated on CTRP-L1000 and Achilles-L1000 datasets (color coded) by calculating Pearson correlation (*y* axis) between predicted and observed values (results from 20 random sub-sampling validations, means ± SD). (**D**) The effects of perturbation time on the predictability of cell viability. Signatures from different time points (3, 6, and 24 h for CTRP-L1000 and 96, 120 and 144 h for Achilles-L1000) were used to train (*x* axis) and test (*y* axis) linear models. Standard models were trained on cell line–perturbation ID data. Size and colors of circles are proportional with the Pearson correlation, which is also labeled in selected cases (mean results from 20 random sub-sampling validations).

To analyze the possible effect of cell viability on the perturbation signatures from LINCS-L1000 dataset, we matched instances from LINCS-L1000 (4) with percent cell viability (data from the Cancer Therapeutics Response Portal (CTRP) (10) and shRNA abundance (measured after lentiviral based pooled RNA interference) data from project Achilles (12). The matching was done based on cell line, perturbation, and, in the case of compounds, concentration (Figure 1A; Materials and Methods section). We removed data points where matching was not possible (e.g. cell lines present in cell viability dataset but not in LINCS-L1000). While percent cell viability and shRNA abundance are different metrics, they are related terms (as both of them are proportional to the number of surviving cells after drug or shRNA treatment), so for simplicity we will refer to both as cell viability hereafter. For perturbation signatures, we included only the actual measured (landmark) genes in this whole study. While in the CTRP and Achilles screen all cell viability values were measured at one time point (72 h and 40 days/16 population doublings, respectively) after perturbation, in the LINCS-L1000 dataset perturbation signatures were measured at different time points. Hence, it is possible to match two different LINCS-L1000 signatures (same compound/shRNA and cell line, but different time points) with the same cell viability value (Figure 1A). Using our matching criteria we were able to compose two datasets: CTRP-L1000 of 18748 matched perturbation signature–cell viability pairs (332 compounds, 48 cell lines, Supplementary Table S1) and Achilles-L1000 of 77230 matched perturbation signature–cell viability pairs (12925 shRNAs, 11 cell lines, Supplementary Table S1).

To explore the main factors behind the perturbation signatures, we performed Principal Component Analysis (PCA) on the signatures of the CTRP-L1000 dataset. While we observed no clustering of signatures in the first two principal component (PC) plane based on cell lines, perturbatogen compounds or perturbation time (Supplementary Figure S1), we found a weak but significant relationship between PC1 and matched cell viability values (Figure 1B, Spearman correlation: −0.278, *P* < 1e-300).

Based on this PCA, we hypothesized that cell viability can be effectively predicted from perturbation signatures. We used linear models (*y = Xβ*) with L2 regularization trained on the L1000 signatures (*X*) and cell viability values (*y*). Using random sub-sampling validation (Materials and Methods), we were able to predict ‘within’ the CTRP-L1000 dataset (Figure 1C) with average Pearson correlation 0.59 (predicted versus observed cell viability, average log10(*p*) < -300) while the performance of ‘within’ Achilles-L1000 prediction models was 0.49 (average log10(*p*) < -300). Furthermore, we were able to predict cell viability ‘across’ the two dataset, predicting cell viability in the CTRP-L1000 dataset with model trained on Achilles-L1000 (average Pearson correlation: 0.33, average log10(*p*) value −246) and with modest performance (average Pearson correlation: 0.19, average log10(*p*) value < -300) in the Achilles-L1000 dataset with model trained on CTRP-L1000. These results suggest that perturbation signatures are associated with cell viability independent of the perturbation agent.

As previously mentioned, LINCS-L1000 dataset contains signatures from different elapsed time between perturbation and measurement. To analyze the effect of this elapsed time on the prediction performance and to select the best datasets for prediction, we split the CTRP-L1000 and Achilles-L1000 datasets based on measurement times (resulting CTRP-L1000-3h, CTRP-L1000-6h, CTRP-L1000-24h, Achilles-L1000-96h, Achilles-L1000-120h and Achilles-L1000-144h datasets, Supplementary Table S1). To further evaluate the performance of signature-based prediction (Figure 1D), we also introduced ‘standard’ models (linear models trained not on signature–cell viability data, but on cell line and perturbation ID–cell viability data, see ‘Materials and Methods’ section for further details). We trained linear models (with L2 regularization) for each time specific dataset, and tested them on the same dataset (‘within’ dataset prediction) and all the other datasets (‘across’ dataset prediction) using random sub-sampling validation. ‘Standard’ models were only used in the ‘within’ dataset setting, as there is no overlap between perturbations for CTRP (drugs) and Achilles (shRNA) datasets, making ‘across’ dataset prediction impossible. While standard models enabled no ‘across’ dataset prediction, we assumed that they show the gold standard for ‘within’ dataset predictions, as they have full information about the used perturbations and cell lines. While all signature-based models performed reasonably well in the ‘within’ dataset setting (Figure 1D diagonal), the CTRP-L1000-24h and Achilles-L1000-96h models reached the best performances in the ‘across’ dataset prediction. These two models also reached comparable performances (0.62 versus 0.66 and 0.51 versus 0.61 Pearson correlation, respectively) with the ‘standard’ models, and were able to make translatable predictions across CTRP and Achilles datasets (which would be impossible for the ‘standard’ models, as discussed previously). Based on this benchmark, we selected CTRP-L1000-24h and Achilles-L1000-96h models for further use in this study.

### Functional genomic analysis of cell death signature

**Figure 2.**
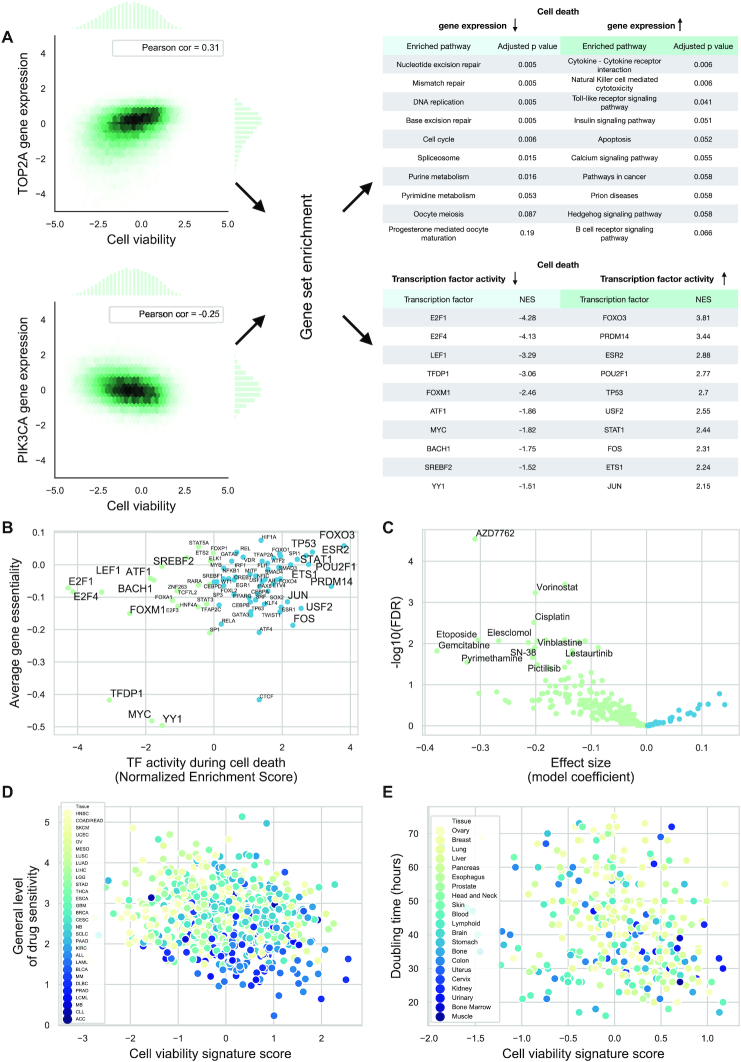
Functional genomics analysis of cell death signature. (**A**) Pearson correlations between gene expression and cell viability values were calculated for the Achilles-L1000-96h dataset for each gene (left, representative examples for PIK3CA and TOP2A). Using these correlation values, KEGG pathway (top right) and Transcription Factor regulon (bottom right) enrichment scores were calculated. (**B**) Association between activity and average Gene Essentiality for transcription factors. Negative Gene Essentiality means the genes are essential (Pearson, *r*: 0.32, *P* = 0.005). (**C**) Volcano plot of cell death signature–drug sensitivity associations from the GDSC dataset. Cell death signature scores were calculated for each cell line using baseline gene expression. A linear model was fit to ln IC50s and signature scores (using tissue type and microsatellite instability as covariates) for each drug. FDR corrected *P* values (*y* axis) for the coefficient of signature scores are plotted against effect size (linear model coefficient) for the signature score parameters. Negative effect size means IC50 values for a given drug are lower (i.e. increased sensitivity) for cell lines with higher cell viability signature scores. (**D**) Associations between cell viability signature score and general level of drug sensitivity (GLDS). GLDS was calculated from GDSC drug sensitivity data. Each point represents a cell line (color coded by tissue type). Pearson correlation: *r* = −0.18 *P* = 5.02e-07. (**E**) Association between cell viability signature score and doubling time. Doubling time (and gene expression for signature score calculation) are from the gCSI dataset. Each point represents a cell line (color coded by tissue type)–Pearson correlation *r* = −0.23, *P* = 2.4e-5.

Effective performance of linear models across compound- and shRNA-based viability datasets suggests that there is some general transcriptomics signature of cell viability. To functionally analyze this cell viability related gene expression signature, we calculated the Pearson correlation coefficient values between gene expression and cell viability (Achilles-L1000-96h dataset) for each gene (representative examples in Figure 2A, left), and performed gene set enrichment analysis using these correlation values as gene level statistics. Using KEGG pathways (20) as gene sets, the most significantly enriched pathways are closely related to cell proliferation and death processes (Figure 2A, top right). We also performed Gene Ontology (GO) enrichment, using biological process GO terms (19). The most significantly enriched GO terms (Supplementary Figure S2A) were also related to cell viability phenotype. Performing the same analysis using data from the CTRP-L1000-24h dataset led to similar results (Supplementary Figure S2).

While KEGG pathway and GO term enrichment focused on the function of genes with cell viability associated expression, we were also interested in the pathways and transcription factors (TFs) regulating these genes. We estimated pathway and transcription factor activities using the PROGENy (5,26) and DoRothEA (21,22) tools, respectively (using the same gene–cell viability correlations as for GO and KEGG enrichment analysis). Some of the most activated (e.g. TP53, FOXO3) and inactivated (e.g. E2F1, FOXM1 and MYC) transcription factors (Figure 2A, right bottom) during cell death are well known regulators of cell death or proliferation. As TFs are main drivers of gene expression changes, we hypothesized that TFs whose activity is correlated with cell viability could be causal factors behind cell death or proliferation. To test this hypothesis, we calculated average gene essentialities across all tested cell lines, for TF coding genes from the Achilles screen, and compared them with the previously calculated transcription factor activities (Figure 2B). The significant correlation (Pearson correlation: 0.32, *P* = 0.005) between these two independently derived metrics suggests that TFs identified by the analysis of the cell viability signature (e.g. MYC, YY1, TFDP1, FOXO3 and ESR2) can be directly involved in growth and survival. The calculated pathway activities from PROGENy also showed an association between pro-survival (e.g. MAPK and PI3K), apoptotic (p53) signaling pathways, and cell viability signature (Supplementary Figure S2E).

To further investigate the functional properties of the cell viability signature, we analyzed the associations between baseline activity of the cell viability signature and GDSC drug sensitivity data (9). Cell viability signature scores were calculated using the Achilles-L1000-96h linear model and baseline gene expression values of the ∼1000 GDSC cell lines. For each of the 266 drugs, we fit a linear model between drug sensitivity (ln IC50 values) and signature score, using tissue type and microsatellite instability status as covariates (according to (5)). Several drugs showed significant (FDR < 0.05 for the coefficient of signature score) associations with the cell viability signature (Figure 2C) including mostly direct cell proliferation inhibitor compounds like CHEK1/2 inhibitor AZD7762, HDAC inhibitor Vorinostat and TOP2 inhibitor Etoposide, where increased CVS is associated with drug sensitivity. To assess the importance of these significant associations, we performed the same analysis with randomized signatures (randomizing gene or sample IDs before fitting the Achilles-L1000-96h linear model) and with ‘single gene’ signatures (using only one of the L1000 genes). Our cell viability signature showed more significant associations than random or single gene signatures (8.0, 4.1 and 9.0 percent of signatures showed a larger or equal number of significant associations with drug sensitivity in the case of randomized gene IDs, sample IDs and single gene expressions, respectively, Supplementary Figure S3A), showing biological relevance of the cell viability signature. We obtained similar results using AUC values from GDSC screen as drug sensitivity metric (Supplementary Figure S3B).

There were a large number of associations between the cell viability signature and cytotoxic (not targeted)/direct proliferation inhibitor compounds. This raised the possibility of the cell viability signature associating with some general features of the cell lines related to drug sensitivity. To investigate this, we compared our signature score with the general level of drug sensitivity metric from (28). General level of drug sensitivity (GLDS) measures drug (and target) independent drug sensitivity of cell lines. We calculated GLDS for GDSC cell lines (Materials and Methods) and found a significant correlation between the cell viability signature score and the GLDS (Pearson correlation: *r* = −0.18 *P* = 5.02e-07, Figure 2D). As GLDS is known to be associated with tissue type ((28) and Supplementary Figure S3C), we wondered whether the CVS score–GLDS correlation is due to the confounding effect of tissue type. To analyze this, we calculated partial correlations between CVS scores and GLDS (using tissue type as covariate, see Materials and Methods) and compared this partial correlation value with partial correlations between GLDS and random or ‘single gene’ signatures. We found that CVS performed better (with 3.6, 2.1 and 5.3 percent of signatures showing smaller partial correlation with GLDS in case of randomized gene IDs, sample IDs and single gene expressions, respectively; Supplementary Figure S3E). Based on recent results suggesting that general drug sensitivity of cancer cell lines is related to division time (29,30), we further analyzed the association between cell viability signature and cell division. We evaluated the gCSI (17) dataset, since baseline gene expression and doubling time were both measured in this study. Signature score showed significant correlation with doubling time (Pearson correlation *r* = −0.23, *P* = 2.4e-5, Figure 2D). Based on the relationship between doubling time and tissue type (Supplementary Figure S3D), we calculated the partial correlation between doubling time and cell viability signature score (using tissue type as a covariate) and compared it with partial correlations for random signatures and single genes. We also found a superior performance of the cell viability signature over single gene and random signatures (0.2, 0.0 and 0.5 percent of signatures showed smaller partial correlation with doubling time, in case of randomized gene IDs, sample IDs and single gene expressions, respectively, Supplementary Figure S3F). This analysis suggests that the cell viability signature is also useful with baseline gene expression values, and shows association with cell division time.

### Signature of cell death as a confounding factor for mechanism of action discovery

One important application of perturbation signatures is to analyze the mechanism of action of drugs (3,4). Compounds that have the same or similar target molecules lead to similar transcriptomic responses. This can help to identify previously unknown targets of compounds. However, toxic compounds with different MoA can lead to a cell death specific signature, which can be incorrectly interpreted as MoA similarity.

**Figure 3.**
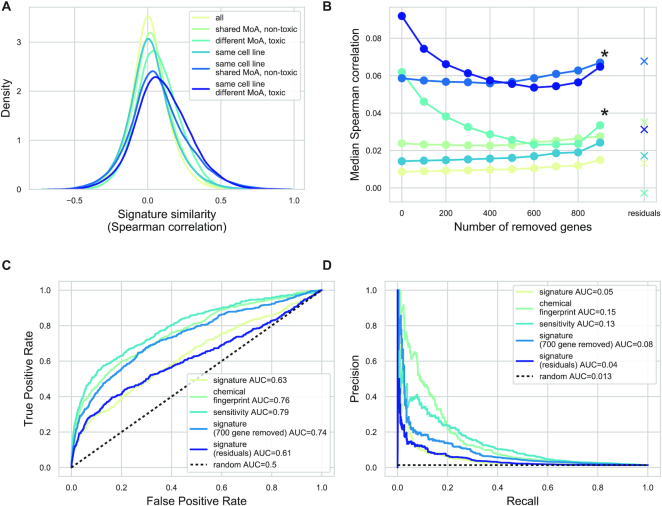
Cell viability signature as a confounding factor for mechanism of action discovery. (**A**) Mechanism of action and cell viability based signature similarity. Spearman correlation (*x* axis) was calculated for pairs of CTRP-L1000-24h signatures with the following constraints (color code): unrelated, nontoxic (cell viability >0.8) compounds with shared MoA and toxic (cell viability <0.8) compounds with different MoA. The Spearman correlation was calculated irrespective of cell line and for signatures from the same cell line. (**B**) The effect of removing cell viability correlated genes on signature similarity. Top *n* (*x* axis) genes with highest absolute Pearson correlation to cell viability were removed from the signatures before similarity calculation. The median Spearman correlations (*y*) axis are shown. The color code corresponds to (A) panel. Similarity for residual gene expression (after regressing out cell viability) was also calculated. *: significant effect (*P* < 0.001, for linear model coefficient) of removing cell death correlated genes (**C** and **D**). The effect of removing cell viability correlated genes on MoA discovery. Average signatures for 327 compounds from CTRP-L1000-24h dataset were calculated using the MODZ method, either using all genes or after removing 700 genes with the highest (absolute) correlation to cell viability. Signature similarity (Spearman correlation) was calculated for each compound pair. For comparison, chemical structure similarity (Tanimoto similarity of chemical fingerprints) and sensitivity profile similarity were also calculated. ROC (C) and Precision Recall curves (D) were used to evaluate the predictive performance of similarity scores on drugs with shared mechanisms of action.

To analyze if this can be a confounding factor to infer mechanism of action, we calculated signature similarity between all signature pairs of CTRP-L1000-24h datasets, using the Spearman correlation as a similarity metric. We focused on the similarities between signatures of nontoxic (defined by cell viability >0.8 for the given cell line, drug and concentration) perturbation pairs with shared MoA (based on (25)) and toxic (cell viability <0.8) perturbation pairs with different MoA (Figure 3A). We calculated similarity irrespective of cell line and for signatures from the same cell line, allowing us to analyze the general and cell line specific nature of signature similarity. Signature pairs of nontoxic perturbations with shared MoA are more similar than random pairs (Medians: 0.023 versus 0.008 and 0.058 versus 0.014, for different and same cell line signatures, respectively, Mann–Whitney *U P* values: <1e-300). More interestingly, signature pairs of toxic perturbations with different MoA are also more similar than random pairs (medians: 0.061 versus 0.008 and 0.091 versus 0.014, for different and same cell line signatures, respectively, Mann–Whitney *U P* values: <1e-300) and similarity between toxic, different MoA signatures was higher than nontoxic, shared MoA signature similarity (Mann–Whitney *U P* values: <1e-300). These results suggest that cell death/toxicity is at least as important factor for signature similarity as mechanism of action, potentially confounding signature-based MoA discovery.

To reduce this unwanted, cell death based signature similarity, we systematically removed the genes with highest absolute Pearson correlation with cell viability and calculated signature similarity based on these reduced signature vectors (Figure 3B). While removing these genes did not strongly affect the MoA-based similarity, it significantly reduced the similarity of toxic signatures (*P* value of the linear model coefficients: 3.2e-05 and 1.6e-04 for different and same cell line signatures, respectively). In contrast, removing genes randomly did not affect signature similarity (Supplementary Figure S4A). We also tried an alternative approach to remove the unwanted effect of cell viability on signature similarity. In this case, we regressed out the effect of cell viability from the gene expression signature before the similarity calculation (see Materials and Methods section for further details), leading to a better performance (decreased similarity for signatures of toxic but unrelated compounds Figure 3B) than raw signatures. In summary, removing the association between cell viability and gene expression (either by removing highly correlated genes or by a regression model) decreased the toxicity based similarity, but did not affected the MoA based similarity.

We also analyzed how signature similarity could predict mechanism of action. For this purpose, we focused on consensus signatures of compounds (calculated using the MODZ method, see Materials and Methods section). We calculated the consensus signature for each compound in the CTRP-L1000-24h dataset, and calculated the pairwise similarity (Spearman correlation) between the consensus signature vectors. We used these similarity values as predicted values for the ROC and Precision–Recall curve (PRC) analyses (Figure 3C and D). The ground truth values were 1 for compound pairs with shared MoA and 0 otherwise. We also compared the performance of signature similarity based MoA predictions with structure and drug sensitivity similarity based MoA predictions, similar to the work of El-Hachem *et al.* (31) (see Materials and Methods section for details). Predictions based on structure and sensitivity outperformed the method based on the full signature (0.76 and 0.79 ROC AUC versus 0.63, respectively, *P* values <1e-12, DeLong test). However, using a reduced signature (by removing 700 genes with the highest absolute correlation to cell viability) led to a comparable performance with structure and sensitivity based methods (0.74 ROC AUC, *P* values 0.14 and 0.0004 versus structure and sensitivity based similarity, respectively, DeLong test). It also led to a clear performance increase when compared to the full signature (*P* < 1e-12, DeLong test). We had similar results for PR curves with PR AUCs of 0.056, 0.153, 0.131 and 0.089 for full signature, structure, sensitivity and reduced signature-based similarity, respectively, while random performance showed a PR AUC of 0.013. In case of MoA prediction, residual signatures did not reach the performance of full signatures (0.61 ROC AUC and 0.04 PRC AUC). Removing different numbers of cell viability correlated genes (Supplementary Figure S4B) had a similar effect to individual signatures, and removing random genes did not strongly affect performance (Supplementary Figure S4C), supporting the confounding nature of cell viability for MoA discovery. As the CTRP dataset uses known anticancer drugs, we hypothesized that cell viability could have a more pronounced effect in this dataset than in the case of general drugs. To analyze our method on a more balanced dataset (with less toxic drugs), we selected all of the compound perturbation signatures from LINCS-L1000 with known target molecules (LINCS-L1000-MoA dataset, 2865 compounds, Supplementary Table S1) (25). For this dataset, we also performed ROC and PR analysis using signature and chemical similarity based MoA prediction (without drug sensitivity data, as it was unavailable). While structure based similarity had better performance in this case also, reduced signature outperformed full signature (ROC AUCs 0.61 versus 0.57 and PRC AUCs 0.024 versus 0.021, Supplementary Figure S4D and E), and more importantly combining the reduced signature with chemical similarity led to the best results (ROC AUC 0.7, PRC AUC 0.088 versus 0.67, and 0.081 for chemical similarity alone), highlighting the importance of transcriptomic signatures in MoA discovery and the confounding nature of cell viability.

### LINCS-L1000 as a cell viability assay

LINCS-L1000 contains a large number of chemical perturbations (which we shall call LINCS-L1000-Chem subset, 21921 compounds, Supplementary Table S1), where most of the used compounds are not known as anticancer drugs. We hypothesized that some of these drugs could have an unexpected, cell line specific anticancer activity that could be identified by predicting cell viability from the perturbation signatures. We used the CTRP-L1000-24h and Achilles-L1000-96h models (based on their top performance, Figure 1D) to predict cell viability for the whole LINCS-L1000-Chem dataset and identified several known and also potentially clinically interesting drugs with cell line specific toxicities.

**Figure 4.**
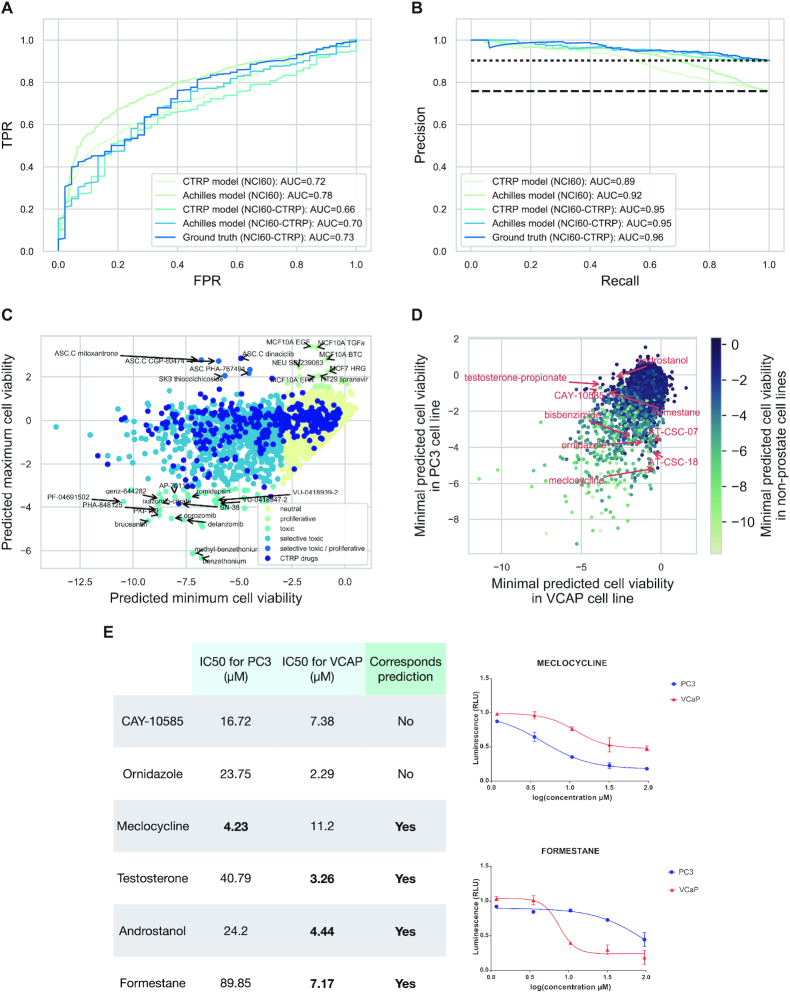
Predictions of cell viability for the whole LINCS-L1000 dataset. (**A** and **B**) ROC and Precision Recall analysis of the prediction performance of linear models on NCI60 data. Cell viability was predicted for the intersection of NCI60 and LINCS-L1000 or for the intersection of NCI60, CTRP and LINCS-L1000 datasets (NCI60 and NCI60-CTRP, respectively) using linear models trained on CTRP-L1000-24h or Achilles-L1000-96h data. Either these predicted cell viability values or the known AUC values from CTRP screen (CTRP AUC) were used to predict the binarised (effective/ineffective in the investigated concentration range) GI50 from NCI60. ROC (A) and PR (B) curves are shown with corresponding AUC values. (**C**) Classification of the compounds from the LINCS-L1000 dataset based on their effect on cell viability. Cell viability was predicted for the LINCS-L1000-Chem dataset (24 h signatures, highest used concentration) using the Achilles-L1000-96h model. The minimum (*x* axis) and maximum (*y* axis) predicted cell viability was plotted for each compound. Compounds were classified as toxic (predicted cell viability < −3) or proliferative (predicted cell viability >1.5) and colored based on toxicity and selectivity (based on maximal and minimal predicted values). Compounds present in the CTRP dataset (known anticancer drugs) were also labeled. For selected compounds the name of the compound and the corresponding cell line is text labeled. (**D**) Cell selective toxicity of LINCS-L1000 compounds in prostate cancer cell lines. Minimal predicted cell viability for VCaP (*x* axis) and PC3 (*y* axis) prostate cancer cell lines was plotted for each compound. Data points are color coded based on the minimal predicted cell viability in nonprostate cancer cell lines. For selected compounds showing selective toxicity in prostate cancer cell lines the name of the compound is text labeled. (**E**) IC50 values of experimentally validated compounds for VCaP and PC3 cell lines. For meclocycline and formestane dose–response curves are also shown (right up and down, respectively), while other full dose–response curves for other compounds are shown in Supplementary Figure S7.

To further evaluate the prediction performance of these linear models, we compared the predicted cell viability values with the results of the NCI60 screen (11). As NCI60 is a discovery screen (most of the drugs used did not have strong anti-cancer activity, Supplementary Figure S5A), it is a realistic benchmark dataset for our LINCS-L1000-Chem predictions. We found an intersection of 583 compounds and 6 cell lines (NCI60-L1000-24h dataset, Supplementary Table S1) between the two screens. We binarized GI50 (50% growth inhibition) results of the NCI60 screen (effective/ineffective anticancer drugs, where ineffective means 50% growth inhibition was not reached in the used concentration range, see Materials and Methods section for further details), and compared them with the predicted cell viability (lowest value for each compound - cell line pair) with ROC and PR curves. We also selected the intersection between LINCS-L1000, NCI60 and CTRP datasets (NCI60-CTRP-L1000-24h dataset, 99 compounds, Supplementary Table S1), where we could compare the performance of the linear models against a ‘ground truth based’ method. In this case, the CTRP drug sensitivity metric (the area under the dose–response curve, AUC) was used to predict the effectiveness in NCI60. ROC and PR analysis (Figure 4A) revealed that the performance of the perturbation signature based models is comparable with the ‘ground truth based’ method (*P* values 0.48 and 0.04 for Achille-L1000_96h and CTRP-L1000-24h models, respectively, DeLong test), and that the Achilles-L1000-96h model performed better than the CTRP-L1000-24h model on the NCI-L1000-24h dataset (AUC = 0.78 versus 0.72, *P* = 1.143e-13, DeLong test). We had similar results when TGI (total growth inhibition) or LC50 (50% lethal concentration) were used as NCI60 drug sensitivity metrics (Supplementary Figure S5B and C), further suggesting the reliable performance of our models to predict cell viability in independent datasets.

As the Achilles-L1000-96h model had the best performance across all benchmarking experiments (Figures 1D and 4A; Supplementary Figure S4D and E), we analyzed the predictions of this model for the whole LINCS-L1000-Chem dataset . We focused on the highest concentration instances of each compound and selected the lowest (most toxic) and highest (least toxic/most proliferative) predicted cell viability for each compound. These lowest and highest predicted cell viability instances came from different cell lines, thus allowing joint-analysis of general and cell-specific compound toxicity. We plotted the lowest and highest predicted cell viability values for each compound against each other, and grouped compounds to toxic and proliferative groups based on arbitrary thresholds (5th and 95th percentiles of cell viability values from the Achilles-L1000 data) of predicted cell viability (Figure 4B). Comparing the maximal tested concentrations of toxic and nontoxic compounds (Supplementary Figure S6) revealed that toxic compounds had slightly higher maximal used concentration (median 10 μM versus 5 μM for toxic and nontoxic compounds, respectively, Mann–Whitney *U* test *P* value < 1e-10), but still lower maximal tested concentration than the ones in a typical cell viability screen (e.g. for CTRP median 66 μM, Mann–Whitney *U* test *P* value < 1e-10 versus toxic group). The known anticancer drugs from the CTRP screen had a high representation in the toxic group (57.9 percent versus 6.8 percent for all compounds, Fisher exact test *P* value: 5.9e-130). In the general toxic group (toxic effect in all screened cell lines), we found proteasome inhibitors (delanzomib, oprozomib), detergent (benzethonium), cell cycle inhibitor (PHA-848125), topoisomerase inhibitor (SN-38), antieukaryotic antibiotic (romidepsin) and plant derivative (bruceantin). Our predictions also revealed the known proliferative effect of epithelial growth factor receptor (EGFR) agonist ligands (EGF, TGFa and betacellulin) in breast cell lines. More interestingly, we identified several cyclin-dependent kinase (CDK) inhibitors (dinaciclib, CGP-60474, PHA-767491) with cell line specific predicted proliferative/toxic effects.

To further analyze the ability of the Achille-L1000-96h model to predict cell line specific compound toxicity, we focused on the two prostate cancer cell lines (PC3 and VCaP) present in the core cell lines of LINCS-L1000 (Figure 4C). Our model predicted several compounds with cell line specific toxicity for these two prostate cancer cell lines, including HIF1A inhibitor CAY-10585, several androgen receptor related compounds (androstanol, testosterone-propionate and formestane) for VCaP, and antibiotics ornidazole and meclocycline for PC3 (Figure 4C, text labeled data points). For these six drugs, with markedly different predicted toxicity in the two prostate cancer cell lines, we performed cell viability measurements (Materials and Methods section). Half inhibitory concentrations (IC50s, Figure 4D and Supplementary Figure S7) showed increased sensitivity of PC3 for meclocycline (4.23 μM versus 11.2 μM for VCaP) and increased sensitivity of VCaP for testosterone, androstanol, and formestane (3.26, 4.44 and 7.17 μM versus 40.79, 24.2 and 89.85 for PC3, respectively), confirming 4 of our 6 predictions. In the case of the two other predictions, we observed low toxicity (ornidazole) and ambiguous results (lower IC50 but also lower maximal toxicity in VCaP cell lines for CAY-10585).

### Drug perturbation signatures as features for machine learning models

As shown in the previous sections, cell viability can be effectively predicted from perturbation transcriptomics data. However, for these predictions, the linear models needed measurements of the perturbation signatures for the investigated cell line–compound pairs. We then asked if it would be also possible to predict cell viability/drug sensitivity for cancer cell lines where the actual perturbation measurement was not performed—a much more challenging task. We reasoned that this could be attempted using drug specific consensus signatures together with cell line specific information to predict drug sensitivity.

**Figure 5.**
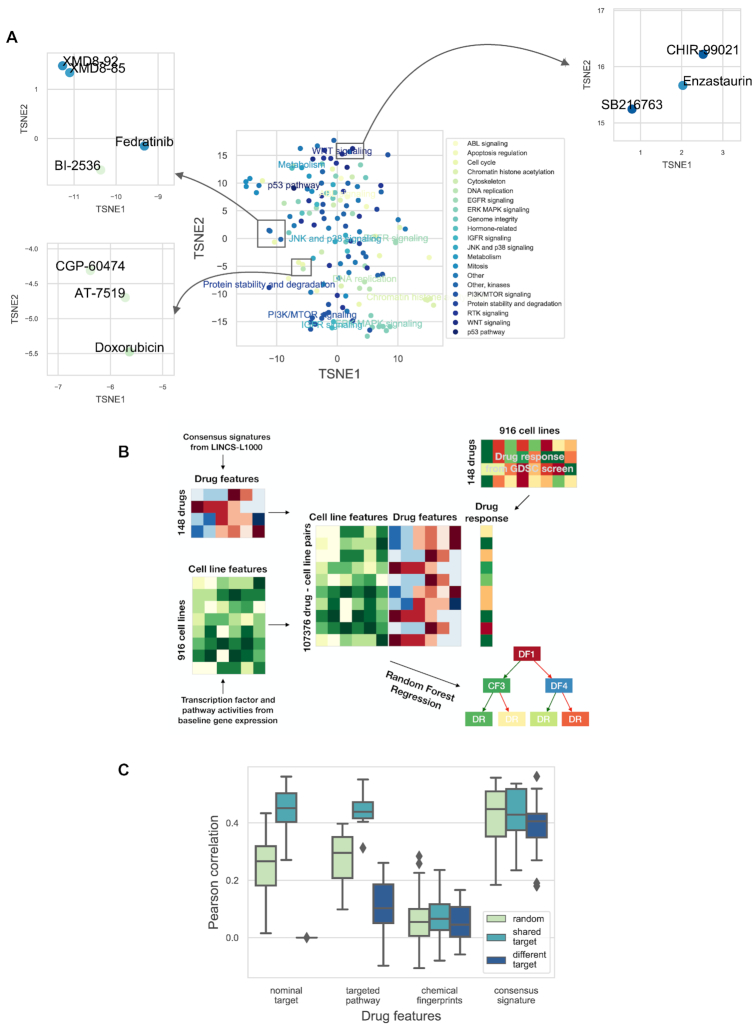
Consensus perturbation signatures for drug sensitivity prediction. (**A**) t-SNE plot (learning rate 80, perplexity 20, number of iterations 1000) of the consensus signatures of GDSC drugs. Data points (intersection of GDSC and LINCS-L1000 drugs, 148 drugs) are colored based on targeted pathway. For selected clusters, the targeted pathway is also text labeled. Inserts: selected clusters of drugs with different pathway annotations. (**B**) A schematic representation of the machine learning pipeline. Cell line specific (tissue type, pathway and transcription factor activity) and drug specific (nominal target, targeted pathway, chemical structure based Extended-Connectivity Fingerprints and consensus signature) feature matrices were created for the cell lines and drugs of the GDSC study. Features were concatenated for each drug–cell line pair where drug response was available in the GDSC screen and this feature matrix was used to train the Random Forest Regression models. (**C**) The results of the machine learning models for drug response AUC predictions. The data were split into training and test sets based on drugs (50–50% percent). Splitting was performed three different ways (color coded): randomly, with the constraint that for each drug in the test set there was a drug with the same nominal target in the training set (shared target), or with the constraint that for each drug in the test set there was no other drug with shared nominal targets in the training set (different target). Different drug specific features (*x* axis) were used by the models. Cell wise average Pearson correlation values are shown as boxplots for the different drug specific features/splitting strategies (results from 20 random subsampling validation).

To achieve this prediction of cell line specific drug sensitivity from a consensus signature, we used machine learning models and applied them to an independent dataset. We chose the GDSC (9) data as it is the largest pharmacogenomic drug screening available. Before evaluating the performance of machine learning models, we analyzed how the consensus drug signatures correspond to the known mechanism of action of the GDSC drugs. Consensus signatures (the MODZ method) were generated for GDSC drugs present in the LINCS-L1000 screen (GDSC-L1000-24h dataset, 148 drugs, Supplementary Table S1). Relationship between consensus signatures were visualized after dimensionality reduction by the t-SNE algorithm (32) (Figure 5A). Some drugs with shared mechanism of action (e.g. MAPK, PI3K and HDAC inhibitors) formed clusters in the t-SNE plane and, even more interestingly, we could observe several clusters formed by unrelated drugs. For example, GSK3 inhibitors CHIR-99021 and SB216763 formed a cluster with PKC inhibitor Enzastaurin, MAPK7 inhibitors XMD8-92 and XMD8-85 formed a cluster with PLK inhibitor BI-2536 and JAK2 inhibitor Fedratinib, while topoisomerase inhibitor Doxorubicin formed clusters with CDK inhibitors, AT-7519 and CGP-60474 (Figure 5A, inserts), which suggests consensus signatures can potentially reveal unknown similarities between anticancer drugs.

We then used Random Forest Regression in multitask setting (predicting drug sensitivity for different cell lines and drugs with the same model) to predict drug sensitivity (area under the dose–response curve, AUC) values from the GDSC, using consensus perturbation transcriptomics signatures from the LINCS-L1000 study as features. To predict drug sensitivity in different cell lines and for different drugs, the Random Forest Regression model required cell line and drug specific features (Figure 5B). For cell line specific features we used tissue type, pathway (5,26) and transcription factor (21,22) activities inferred from baseline gene expression (see Materials and Methods section for further details), while consensus perturbation signatures were used as drug specific features. For benchmarking, we used other types of drug specific features (see Materials and Methods section for further details): nominal target of the drug, targeted pathway of the drug and chemical structure based Extended-Connectivity Fingerprints (ECFP-like), to compare the performance of signature based features with these.

We focused on the prediction of drug sensitivity for new (model unknown) drugs. To do this, we split the GDSC dataset in half, based on the drugs (i.e. half of the drugs were in our training, the other half in the test set). We used three different splitting schemes: random, shared target and different target. In shared target splitting, each drug in the test had a corresponding pair in the training set with the same nominal target, while in different target splitting the drugs in the training and test sets had strictly different nominal targets (see Methods for further details). We evaluated prediction performance by calculating the Pearson correlation between predicted and observed drug sensitivity (dose response AUC) values for each cell line (Figure 5C). In the shared target setting, nominal target, targeted pathway and consensus signature based features had similar performance (mean Pearson correlations: 0.44, 0.44 and 0.42, respectively, ANOVA *P* value for used features: 0.68). Not surprisingly nominal target based drug features were not useful in the case of different target sampling (*P* value from one sample *t*-test with 0 population mean: 0.06), while consensus signature based features outperformed targeted pathway features in this case (mean Pearson correlations: 0.38 and 0.10 respectively, *P* value from paired *t*-test: 7.1e-10). In summary, consensus signature based drug features for machine learning models had better performance than current gold standard drug specific features like nominal target or target pathway, and allowed cell line specific prediction of drug sensitivity. We predicted AUC as drug sensitivity metric in this case, as differences between mean IC50 values for different drugs could have been a confounding factor in our predictions. Nevertheless using (ln) IC50 as a drug sensitivity metric resulted in a similar performance (Supplementary Figure S8).

## DISCUSSION

In this paper, we integrated recent large-scale functional and pharmacogenomic studies to analyze the effect of cell viability on perturbation transcriptomics signatures. Integrating gene expression profiles with phenotypic data (7,33) and drug effect (3,34) have been performed in recent years. However, the association between gene expression and cell viability has not been systematically investigated. We found that cell viability (i.e. cell death and cell proliferation) has a major contribution to the perturbation signatures. While the association between cell viability phenotype and transcriptional signatures enables efficient prediction of cell viability values from perturbation signatures, it can also mask the compound specific transcriptional signal, thus confounding discovery of mechanism of action. By analyzing cell viability signature, we also found transcription factors with causal roles in cell death and proliferation, and found an association between cell viability signature and division time.

Using perturbation metadata from the LINCS-L1000, CTRP and Achilles projects, we composed the largest matched cell viability–perturbation signature dataset (to our knowledge) with >15 000 compounds and >75 000 shRNA treatments. Principal Component Analysis of the CTRP-L1000 dataset revealed that the first PC (explaining 9% of total gene expression variance) is associated (Figure 1B) with cell viability. The cell line and perturbation independent nature of PC1 was already described in one of the original LINCS-L1000 manuscripts (14), and it was speculated that it was connected to some general biological effects, but it was not explicitly analyzed previously. Also, a recent analysis (13) compared perturbation signatures with cell viability. Jung *et al.* matched perturbation signatures from the LINCS-L1000 screen with corresponding cell growth inhibition (cell viability) values from an earlier version of the GDCS with 639 cell line–compound pairs (35) and used these data to identify essential gene signatures. Our study focused on the predictive value of perturbation signatures, the confounding effect of cell viability on signature similarity and mechanism of action identification, and the functional genomic analysis of the identified cell death signature.

Based on this association between cell viability and perturbation signatures, we were able to predict cell viability from the gene expression data. Most importantly, linear models trained on drug perturbations (CTRP data) were able to predict cell viability after shRNA treatment (Achilles data), and vice versa (Figure 1C and D). While ‘standard models’, using perturbation and cell line information, were also able to perform effectively within a given dataset (CTRP or Achilles), the efficacy ‘across’ dataset prediction is unique for the perturbation signature based models. Also, several studies investigated the predictability of drug sensitivity (9,36,37) and gene essentiality (38) with good results; however, translatable prediction was not attempted among these different, yet related phenotypes. There could be two main reasons for the efficacy across dataset prediction performance of our methods. First, that the models could learn the drug/shRNA specific changes in signatures and utilize the similarities between signatures to predict cell viability across different perturbations. The second possibility is that there is a specific cell death signature, independent of the original perturbation agent, and linear models learn this signature. Our functional genomic analysis (Figure 2 and Supplementary Figure S2) suggests the latter. We also analyzed how the elapsed time between perturbation and transcriptomic measurements affects the predictability of cell viability. While the two best performing models (CTRP-L1000-24h and Achilles-L1000-96h) have the largest amount of data available (Supplementary Table S1), the poor performance of 3- and 6-h models also suggest that the cell viability related gene expression changes are only observable after longer perturbations. Importantly, our models were trained on transcriptomic data from the LINCS project and cell viability data from the CTRP and Achilles projects and were evaluated on the NCI60 cell viability data (Figure 4A) i.e. we used data from four different sources. The effective performance of the models across these different studies suggests the underlying biological phenomenon is robust, and also provides a step to help address the challenge of translating models across drug sensitivity screens (39,40).

Using these models, we were able to predict cell viability for the whole compound perturbation part of the LINCS-L1000 study. We were able to identify not only well known general toxic compounds like detergents and proteasome and topoisomerase inhibitors, but also compounds leading to proliferation like EGF in breast cancer cell lines (Figure 4B). Most interestingly, several CDK inhibitors had cell line specific toxic or proliferative effects, where the proliferative effect was observed in adipocyte stem cells (ASCs). While CDK inhibitors can uncouple cell cycle from apoptotic function (41), so the proliferative effect can be a false positive, some experimental evidence also suggests that CDKs can have a paradoxically proliferative effect in stem cells (42). We also analyzed cell line specific predictions in prostate cancer cell lines VCaP and PC3, and performed experimental validations for six compounds showing marked differences of toxicities between these two cell lines. We found that several androgen receptor signaling related compounds (like androstanol and testosterone-propionate) have selective toxic effects in VCaP cell lines. This paradoxically toxic effect of androgen receptor agonists have been reported in another castration resistant prostate cancer model (43). Our results show that sensitivity of different prostate cancer cell lines can be markedly different for androgen treatment, suggesting androgens as therapeutic options in selected cases of metastatic disease (44). We also observed selective toxicity of meclocycline in PC3 cell lines. As meclocycline is an antibacterial antibiotic, it could also be used as a potential treatment with a low adverse effect profile in prostate cancer. Interestingly, meclocycline is a tetracycline antibiotic, a group of drugs known to have a metalloprotease inhibitor effect (45). Matrix metalloproteinases were recently described as potentially important molecules and drug targets in prostate cancer (46). These *in vitro* validations suggest that the predicted cell line selective compounds can be useful anticancer drugs. Further studies, in particular *in vivo*, are needed to confirm that this is the case in a clinical setting.

While our models were able to predict cell viability from the actual measured perturbation signatures, one can argue that, in this case, the experiment is already performed, and if somebody is interested in cell viability, testing viability is simpler and cheaper than perturbation transcriptomics profiling. Yet, our machine learning predictions on the GDSC dataset showed that using consensus signatures (delivered from a small number of core cell lines) as features for machine learning models allows the prediction of drug sensitivity in new cell lines as well. Our results showed that the consensus signature outperforms gold standard features like nominal targets, targeted pathways and chemical fingerprints (Figure 5C) and allows prediction of drug sensitivity for new drugs (unrelated to the ones present in the training set). Also, our models can be used to predict cell viability in previously performed gene expression studies, where cell viability data were not measured.

Clustering of the GDSC drugs based on the consensus signatures (Figure 5A) also revealed important information regarding their mechanism of action. While some drugs with the same MoA (e.g. ERK/MAPK inhibitors and PI3K/MTOR inhibitors) formed tight clusters, we also observed clusters of seemingly unrelated drugs. The similarity of the PKC inhibitor Enzastaurin’s signature to GSK inhibitors’ have been reported in the original LINCS-L1000 study (4), while the connection between CDK inhibitors and Doxorubicin have also been described previously (3). We also observed a cluster composed of XMD8-92, XMD8-85, BI-2536 and Fedratinib (MAPK7, MAPK7, PLK and JAK2 inhibitors, respectively). Recent experiments support that all of these drugs have a common BET inhibitor effect (47,48), likely responsible for the signature similarity.

While the association of perturbation signatures with cell viability enables effective prediction, it can also be a confounding factor for mechanism of action discovery. The similarity between toxic compounds with different MoA is comparable to the similarity among nontoxic compounds with the same MoA (Figure 3A), which can negatively influence MoA discovery (Figure 3C and D). Importantly, removing genes with high absolute correlations with cell viability helps to overcome this problem (Figure 3B and Supplementary Figure S4B), and can help to analyze the results of perturbation transcriptomic signatures more rigorously. We also used signature similarity based MoA prediction on a larger, more diverse dataset (LINCS-L1000-MoA), where, although generally our results were weaker, removing cell viability correlating genes also led to an increased prediction performance (Supplementary Figure S4D and E). While chemical structure based similarities had the superior prediction performance (similar to (31)), it is important to mention that known MoA is mostly based on the on-target activity of drugs (which can be closely associated with chemical structure), but signature based similarity can help to identify off-targets of drugs. While our work focused on using signature, structure and sensitivity profile based similarities independently, recent works (31,49) used the fusion of different similarities to reach optimal MoA predictions. Since removing cell viability correlated genes significantly improved our MoA predictions, using it together with these advanced similarity fusion techniques could further boost MoA prediction in the future.

The functional genomic analysis of the identified cell viability signature also gave us insight into the mechanism of cell death and proliferation. Comparing transcription factor activities and Gene Essentialities (Figure 2B), we identified several transcription factors potentially involved in cell proliferation and cell death (Figure 2A and B). MYC (50) and YY1 (51) are known, promising drug targets, additionally, our analysis also highlighted the TFDP1 transcription factor as a potential drug candidate. The transcription factors showing increased activity during cell death are well known (like TP53 and FOXO3) or emerging (like ESR2 (52)) regulators of apoptosis. We also identified associations between cell viability signature scores and doubling time (Figure 2E) suggesting that, using our models, cell viability related information could also be predicted from baseline (not perturbed) gene expression data. It is important to mention that while doubling time shows associations with the tissue type of tumor cell lines, cell viability signature associated with doubling time independent of the tissue type.

The linear models and pre-calculated predicted cell viability values can be an important resource for further studies working with perturbation gene expression signatures. All of our results are publically available with CEVIChE (CEll VIability Calculator from gene Expression, https://saezlab.shinyapps.io/ceviche/), an R Shiny application, for further analysis. CEVIChE can be also applied to predict cell viability from existing and future signatures. The application can be explored with an example dataset coming from (53). Furthermore, recent methods have been developed to predict unmeasured perturbation gene expression (54) for which our cell viability prediction methods could also potentially be utilized. Also, our results suggest that removing cell death correlating genes from the gene expression signature can help to better interpret the similarities between signatures and identify mechanism of action. Furthermore, drug sensitivity prediction with machine learning models is an important area of current research, and our results (together with other recent works (55)) highlight the importance of using perturbation signatures as features in this field. Finally, while we solely analyzed cell viability for cellular phenotype, the methods presented here could be used in the context of other perturbation–phenotypic measurement studies as well.

## DATA AVAILABILITY

All analysis code is available at https://github.com/bence-szalai/Cell-death-signatures. Our R Shiny application, CEVIChE (CEll VIability Calculator from gene Expression, https://saezlab.shinyapps.io/ceviche) allows the interactive analysis of our results and also the prediction of cell viability in new gene expression samples.

## Supplementary Material

gkz805_Supplemental_File
