# Supplementary material for: Signatures of cell death and proliferation in perturbation transcriptomics data—from confounding factor to effective prediction
Source: Nucleic Acids Res. 2019 Sep 25;47(19):10010–26. doi: 10.1093/nar/gkz805 (PMC6821211; doi:10.1093/nar/gkz805)

Signatures of cell death and proliferation in perturbation transcriptomics data - from confounding factor to effective prediction

Supplementary Material

Bence Szalai[1,2,*], Vigneshwari Subramanian[1], Christian H. Holland[1,3], Róbert Alföldi[4], László G. Puskás[4], Julio Saez-Rodriguez[1,3,*]

[1] RWTH Aachen University, Faculty of Medicine, Joint Research Centre for Computational Biomedicine (JRC-COMBINE), Aachen, Germany

[2] Semmelweis University, Faculty of Medicine, Department of Physiology, Budapest, Hungary

[3] Heidelberg University, Faculty of Medicine and Heidelberg University Hospital, Institute of Computational Biomedicine, Bioquant, 69120 Heidelberg, Germany

[4] Astridbio Technologies Ltd., Szeged, Hungary

* Corresponding author: julio.saez@bioquant.uni-heidelberg.de
szalai.bence@med.semmelweis-univ.hu

Supplementary Figure legends

Supplementary Figure 1 - Clustering of L1000 signatures based on different factors

Principal Component Analysis (PCA) was performed on the perturbation signatures from the CTRP-L1000 dataset. Each point represents a unique cell line - compound - concentration - perturbation time instance. Points are colored according to cell lines (A), compound used for perturbation (B), perturbation time (C), and cell viability (E). Only selected compounds and cell lines (with the largest number of data points) are color labelled. For cell viability based clustering we selected 0.8 as the threshold for toxic / non-toxic clusters based on the histogram (D) of cell viability values (~2 SD below mean based on Gaussian Mixed model). We performed average silhouette analysis using the different clustering factors (F).

Supplementary Figure 2 - Enrichment and pathway activity analysis of genes showing correlated expression with cell viability

Pearson correlations between gene expression and cell viability values were calculated for the CTRP-L1000-24h dataset (A, C, D and F) and for the Achilles-L1000-96h dataset (B, E) for each gene. Using these correlation values, KEGG pathway (A), GO term (B, C), Transcription Factor regulon (D) enrichment scores, and PROGENy pathway activity scores (E, F) were calculated.

Supplementary Figure 3 - Significance of cell viability score associations

(A) The distribution of randomised signature - drug sensitivity associations. Associations between drug sensitivity (IC50) and randomised signature scores and single gene signatures were calculated using linear models (IC50 = f(score, tissue type, MSI)). The histograms show the number of significant (FDR<0.05 for score coefficient) drug - signature score associations, the dotted line shows the number of significant associations for the real model, and the proportion of random signatures showing higher number of significant associations than the real model is text labeled. To create randomised signatures, the Achilles-L1000-96h model was fitted with shuffled genes (left) or sample (middle) labels. Single gene signatures (right) used the expression of each of the L1000 genes. (B) Correlation between effect sizes (left) and log10 p values (right) from the linear models using IC50 or AUC as drug sensitivity metrics. (C) Violin plots for general level of drug sensitivity GLDS) distribution based on tissue type from GDSC data. (D) Violin plots of the division time distribution based on tissue type from gCSI data. (E) The distribution of randomised signature - GLDS partial correlations. Signature scores were calculated for GDSC cell lines using gene wise (left), sample wise (middle), randomised models or single gene expressions (right). The partial correlation between GLDS and signature score (using tissue type as covariate) was calculated. The dotted line shows the partial correlation for the real model, and the proportion of random signatures

showing a lower partial correlation than the real model is text labeled. (F) The distribution of randomised signature - doubling time partial correlations. The signature scores were calculated for gCSI cell lines using gene wise (left), sample wise (middle), randomised models or single gene expressions (right). The partial correlations between doubling time and signature score (using tissue type as covariate) were calculated. The dotted line shows partial correlation for the real model, and the proportion of random signatures showing lower partial correlation than the real model is text labeled.

Supplementary Figure 4 - Effects of cell viability on MoA discovery

(A) Removing random genes did not affect signature similarity. Random n (x axis) genes were removed from the signatures before similarity calculation. Median Spearman correlations (y) axis are shown (from random 10 experiments). (B) Comparison of ROC and PR curves. Average signatures for 327 compounds from the CTRP-L1000-24h dataset were calculated using the MODZ method, either using all genes or after removing different number of genes with the highest (absolute) correlation with cell viability. Signature similarity (Spearman correlation) was calculated for each compound pair. ROC and PR curve AUCs for MoA prediction are plotted. (C) Removing random genes does not affect MoA discovery. Random n genes (color code) were removed from signatures before similarity calculation. MoA discovery was evaluated by ROC (x axis) and PR curve (y axis) AUCs. Mean +/- SD for 10 experiments. (D-E) The effect of removing cell viability correlated genes on MoA discovery. Average signatures for 2865 compounds from the LINCS-L1000-MoA dataset was calculated using the MODZ method, either using all genes, or after removing 700 genes with the highest (absolute) correlation with cell viability. Signature similarity (Spearman correlation) was calculated for each compound pair. For comparison, chemical structure similarity (Tanimoto similarity of chemical fingerprints) was also calculated. ROC (D) and Precision Recall curves (E) were used to evaluate the predictive performance of similarity scores on drugs with shared mechanism of action. In E (x axis) shows curves between 0 and 0.2 for better interpretability.

Supplementary Figure 5 - Model validation in NCI60 dataset

(A) Distribution of effective and ineffective drugs in the NCI60-L1000-24h dataset. Delta concentration was defined as NCI60 the sensitivity metric (GI50, TGI or LC50, from left to right) minus maximal used concentration. Delta concentration 0 indicates an ineffective drug for the investigated cell line (as the drug does not have an effect in the used concentration range). (B,C) ROC and Precision Recall analysis of the prediction performance of linear models on NCI60 data. Cell viability was predicted for the intersection of NCI60 and LINCS-L1000 and for the intersection of NCI60, CTRP, and LINCS-L1000 datasets (NCI60-L1000-24h and NCI60-CTRP-L1000-24h respectively) using linear models trained on CTRP-L1000-24h or Achilles-L1000-96h data. Either these predicted cell viability values or the known AUC values from the CTRP screen (CTRP AUC) were used to predict the binarised (effective / ineffective

in the investigated concentration range) TGI (B) and LC50 (C) values from NCI60. ROC (left) and Precision Recall curves (right) were used to evaluate prediction performance.

Supplementary Figure 6 - Maximal tested concentrations of drugs in LINCS-L1000

Violin plots of maximal tested concentrations of non-toxic and toxic compounds from LINCS-L1000 screen and anti-cancer drugs from CTRP screen. LINCS-L1000 compounds were classified as non-toxic / toxic based on the Achilles-L1000-96h model.

Supplementary Figure 7 - Experimental validation of prostate cancer cell line specific compound toxicity

(A-F) Dose response curves for experimentally tested compounds in PC3 and VCaP cell lines. Cell viability was measured in triplicates after 48 hours incubation with tested compounds. Calculated IC50 values (GraphPad Prism) are shown in the inserts.

Supplementary Figure 8 - Machine learning predictions for GDSC IC50

The results of the machine learning models for IC50 prediction. The data was split into training and test sets based on drugs (50-50% percent). Splitting was performed 3 different ways (color code): randomly, or with constraint that for each drug in a test set there was a drug with the same nominal target in the training set (shared target), or with the constraint that for each drug in a test set there were no other drugs with shared nominal targets in the training set (different target). Different drug specific features (x axis) were used by the models. Cell wise average Pearson correlation values are shown as boxplots for the different drug specific features / splitting strategies (results from 20 random sub-sampling validation).

	Data type	Data points	Cell lines	Compounds	shRNAs	Time points
**LINCS-L1000**	signature	591697	98	21299	18493	12
**CTRP**	cell viability	6171005	887	545	0	1
**Achilles**	cell viability	42893983	501	0	108718	1
**CTRP-L1000**	matched	18748	48	332	0	4
**Achilles-L1000**	matched	77230	11	0	12925	8
**CTRP-L1000-3h**	matched	1100	5	43	0	1
**CTRP-L1000-6h**	matched	7878	46	288	0	1
**CTRP-L1000-24h**	matched	9765	18	327	0	1
**Achilles-L1000-96h**	matched	57639	10	0	10733	1
**Achilles-L1000-120h**	matched	11431	2	0	11366	1
**Achilles-L1000-144h**	matched	7773	3	0	4180	1
**LINCS-L1000-MoA**	signature	149294	82	2865	0	4
**LINCS-L1000-Chem**	signature	320694	83	21921	0	8
**NCI60**	cell viability	3016553	159	52578	0	1
**NCI60-L1000-24h**	matched	2160	6	583	0	1
**NCI60-CTRP-L1000-24h**	matched	466	6	99	0	1
**GDSC-L1000-24h**	signature	21011	41	148	0	1

Supplementary Table 1. Descriptive statistics of the used datasets. *The table includes data type (perturbation signature, cell viability or matched), number of data points, number of cell lines, number of compounds (small molecules or biologicals), shRNAs, and time points (elapsed time between perturbation and measurement) for each used dataset.*

Supplementary Figure 1

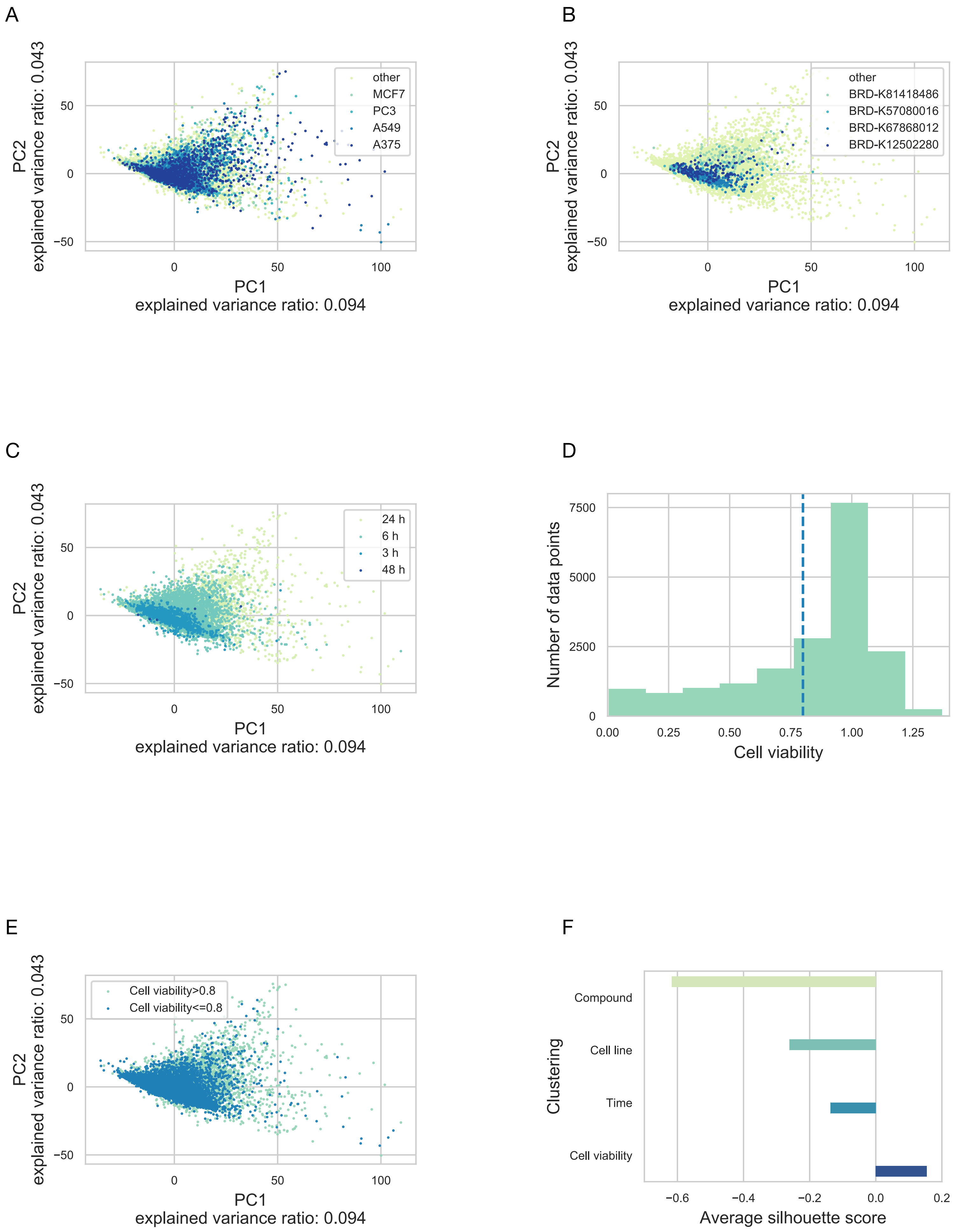

Supplementary Figure 2

A

Cell death

Enriched pathway (gene expression ↓)	Adjusted p value	Enriched pathway (gene expression ↑)	Adjusted p value
DNA replication	0.004	Lysosome	0.014
Mismatch repair	0.004	Natural Killer cell mediated cytotoxicity	0.029
Cell cycle	0.007	B cell receptor signaling pathway	0.029
Base excision repair	0.008	Leishmania infection	0.045
Nucleotide excision repair	0.008	Toll-like receptor signaling pathway	0.067
Progesterone mediated oocyte maturation	0.008	Insulin signaling pathway	0.067
Lysine degradation	0.165	Cytokine - Cytokine receptor interaction	0.067
Huntingtons disease	0.171	Prion diseases	0.067
Oocyte meiosis	0.171	Chemokine signaling pathway	0.067
Purine metabolism	0.216	T cell receptor signaling pathway	0.084

B

Cell death

Enriched GO term (gene expression ↓)	Adjusted p value	Enriched GO term (gene expression ↑)	Adjusted p value
Mitotic recombination	0.007	Positive regulation of response to stimulus	0.02
Regulation of centrosome cycle	0.007	Cellular response to organic substance	0.02
Cell cycle phase transition	0.007	Response to oxygen containing compound	0.02
Transcription coupled nucleotide excision repair	0.007	Response to external stimulus	0.02
DNA repair	0.007	Regulation of multicellular organismal development	0.02
Mitotic nuclear division	0.007	Defense response	0.02
Coenzyme metabolic process	0.007	Anatomical structure formation involved in morphogenesis	0.02
DNA recombination	0.007	Angiogenesis	0.02
DNA replication	0.007	Regulation of ossification	0.02
DNA dependent DNA replication	0.007	Inflammatory response	0.02

C

Cell death

Enriched GO term (gene expression ↓)	Adjusted p value	Enriched GO term (gene expression ↑)	Adjusted p value
Cell cycle	0.004	Inflammatory response	0.039
Cell cycle process	0.004	Response to bacterium	0.039
Mitotic cell cycle	0.004	Anatomical structure formation involved in morphogenesis	0.039
Chromosome organization	0.004	Defense response	0.039
Cellular response to DNA damage stimulus	0.004	Response to lipid	0.039
Regulation of mitotic cell cycle	0.004	Positive regulation of multicellular organismal process	0.039
Protein modification by small protein conjugation	0.004	Regulation of immune system process	0.039
DNA metabolic process	0.004	Negative regulation of cell communication	0.039
Cell division	0.004	Negative regulation of response to stimulus	0.039
Organelle fission	0.004	Response to external stimulus	0.039

D

Cell death

Transcription factor (Transcription factor activity ↓)	NES	Transcription factor (Transcription factor activity ↑)	NES
E2F4	-4.22	FOXO3	3.68
E2F1	-3.89	SMAD4	3.65
TFDP1	-3.61	TP53	3.47
LEF1	-2.77	SMAD3	3.22
ATF1	-2.57	ESR1	3.07
FOXM1	-1.79	ESR2	3.06
MYC	-1.75	SREBF1	2.75
TFAP2C	-1.59	TWIST1	2.74
FOXA1	-1.09	SRF	2.61
YY1	-1.08	POU2F1	2.59

E

Cell death

PROGENy pathway (Pathway activity ↓)	Pathway activity (z score)	PROGENy pathway (Pathway activity ↑)	Pathway activity (z score)
MAPK	-4.33	JAK-STAT	3.26
EGFR	-2.94	p53	1.99
PI3K	-2.92		
Estrogen	-2.36		
Hypoxia	-2.29		

F

Cell death

PROGENy pathway (Pathway activity ↓)	Pathway activity (z score)	PROGENy pathway (Pathway activity ↑)	Pathway activity (z score)
MAPK	-3.77	p53	3.09
PI3K	-3.15	NFkB	2.38
Estrogen	-2.93	TNFa	2.24

Supplementary Figure 3

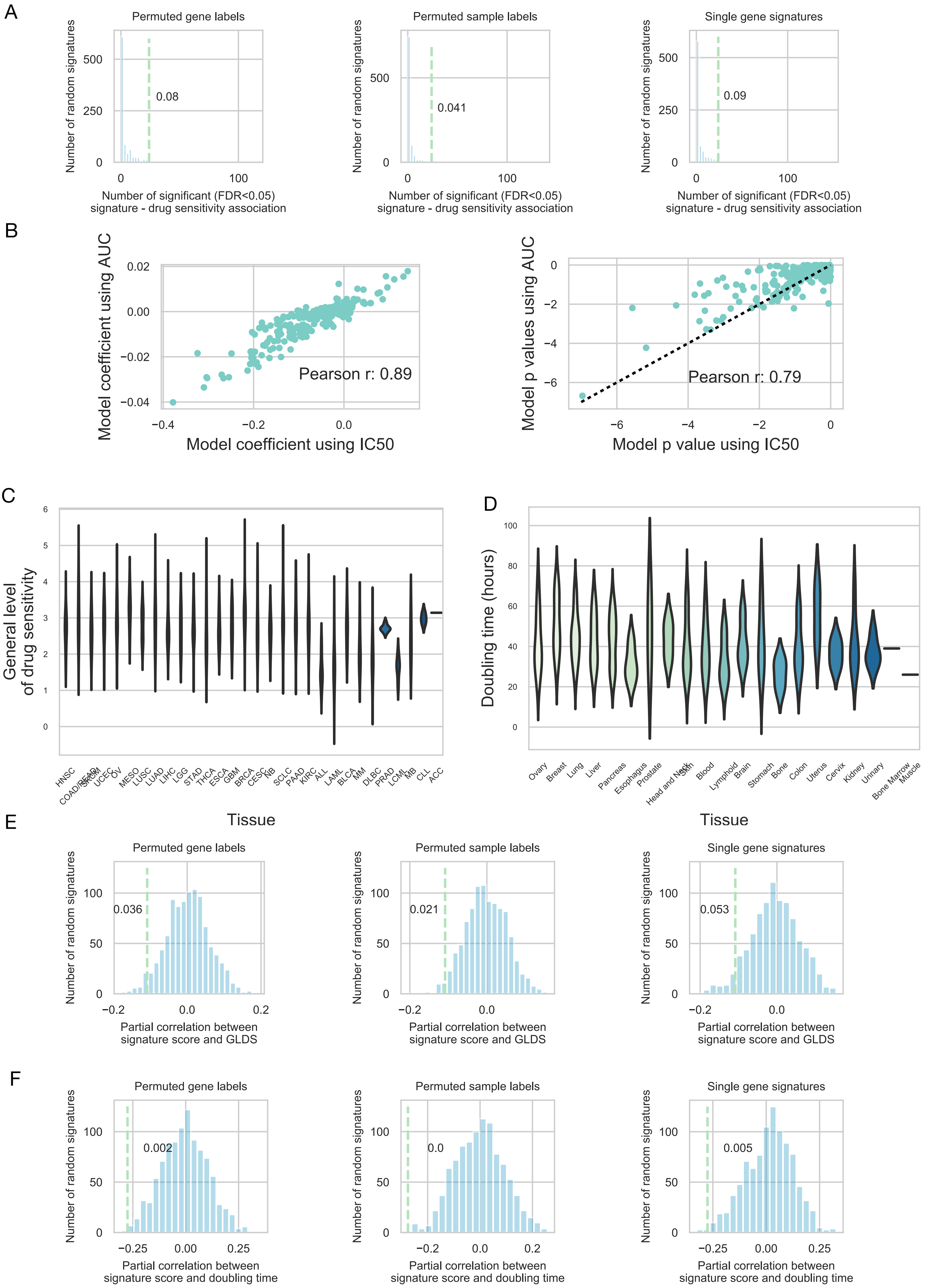

Supplementary Figure 4

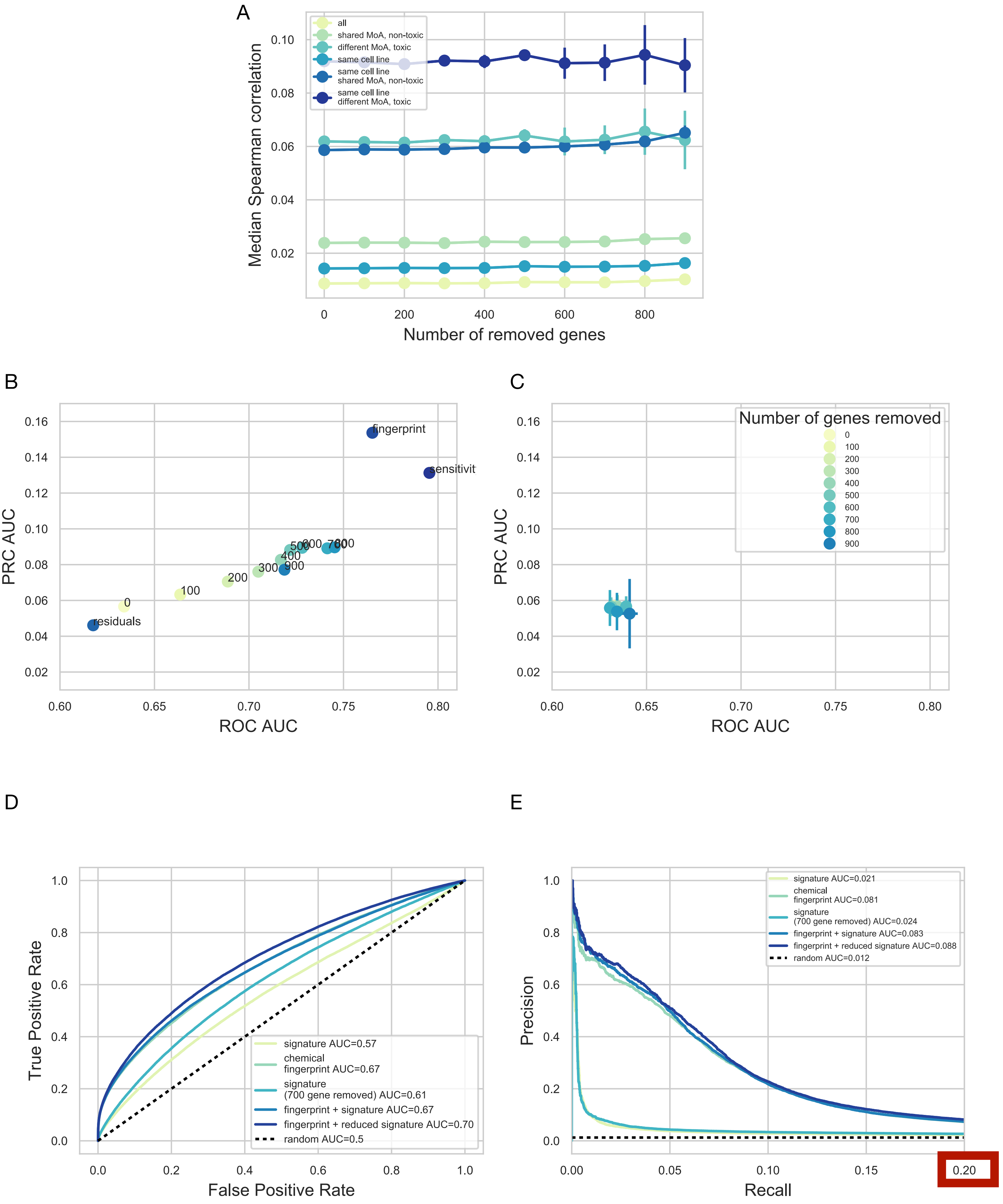

Supplementary Figure 5

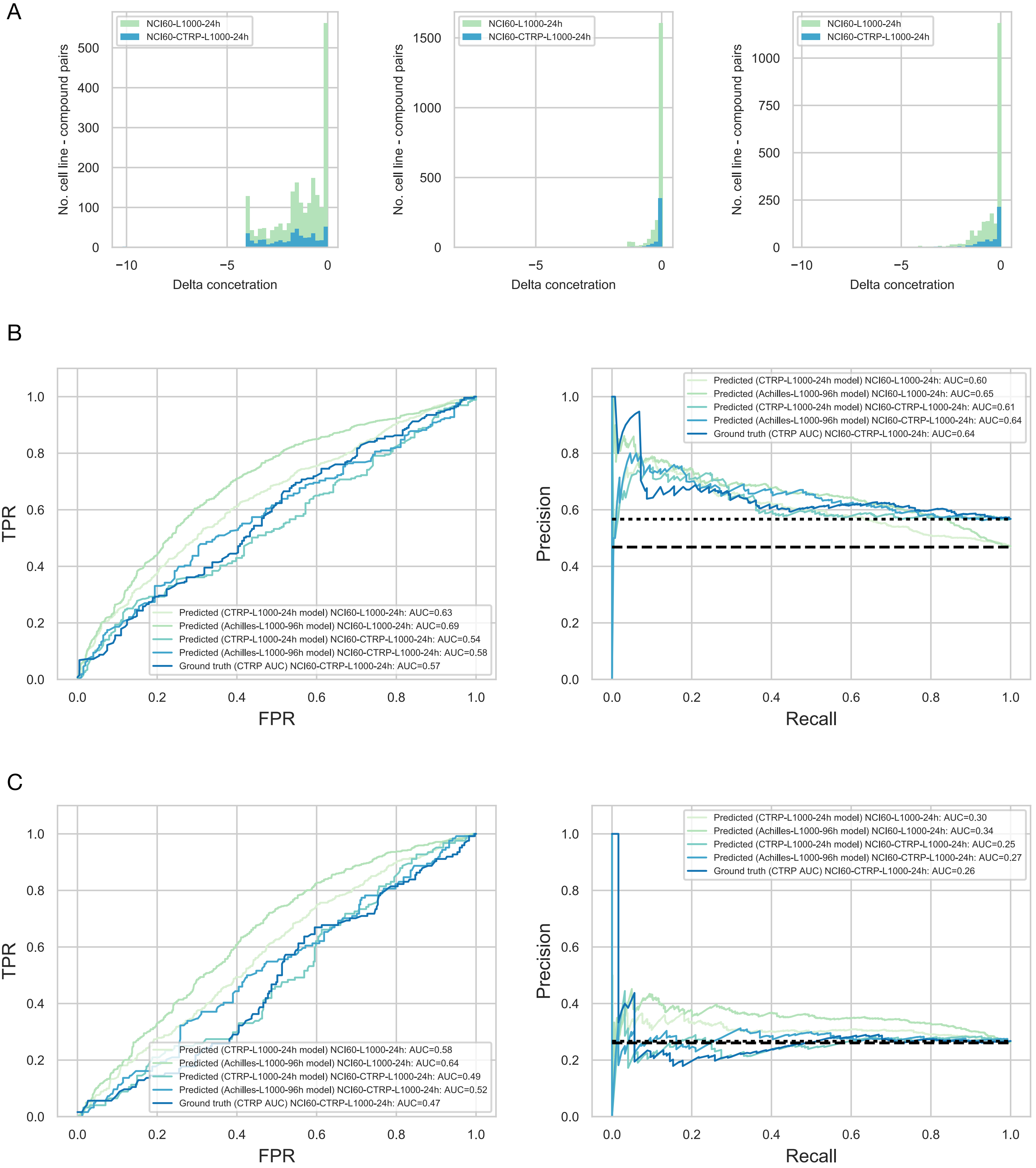

Supplementary Figure 6

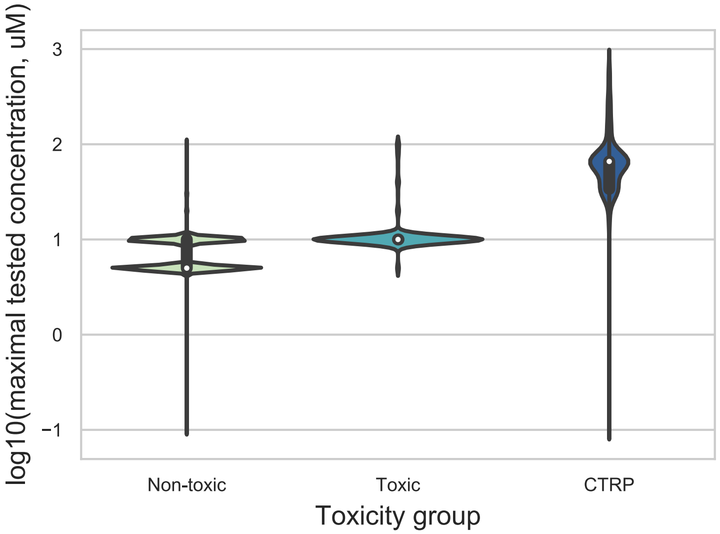

Supplementary Figure 7

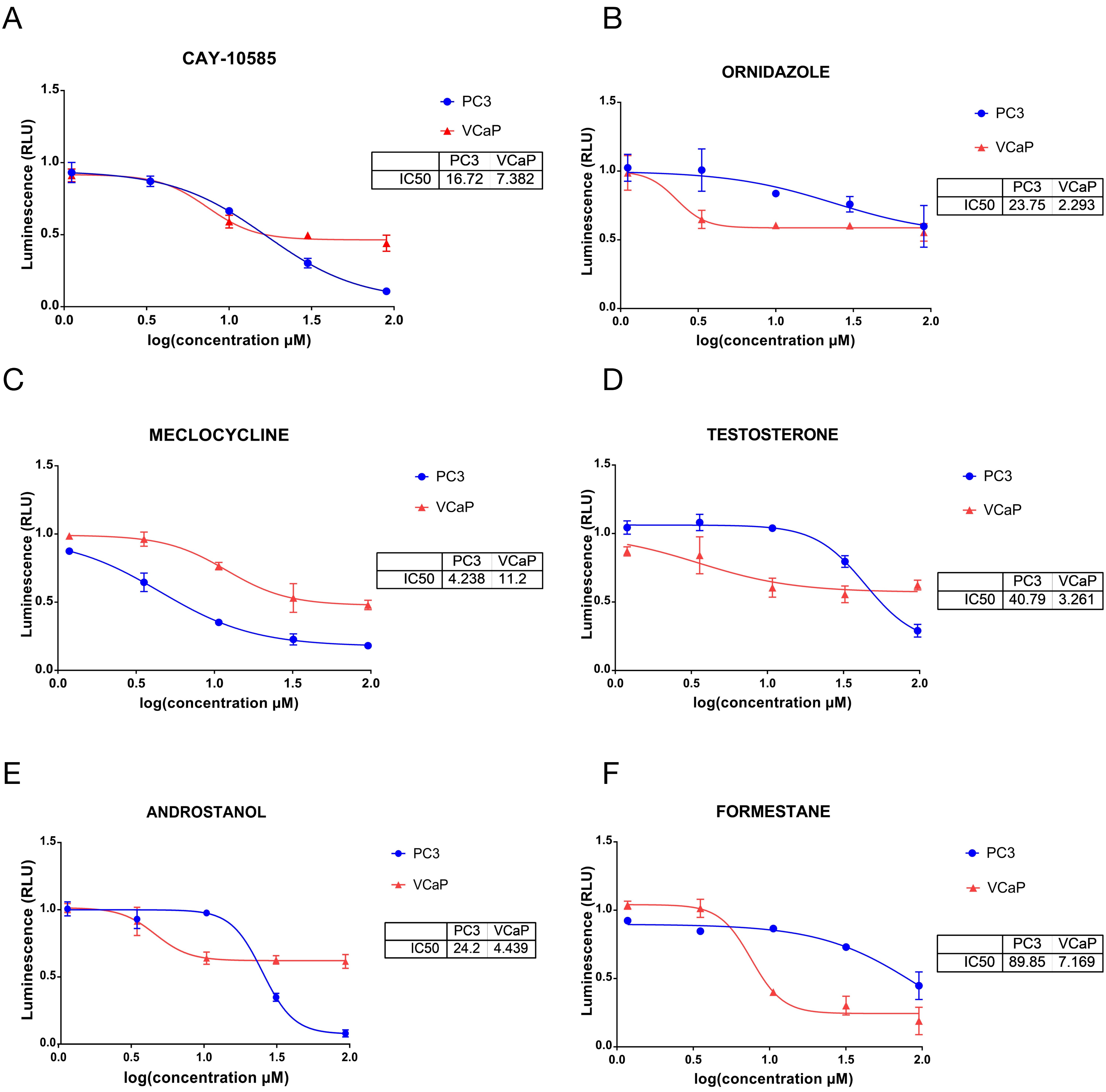

Supplementary Figure 8

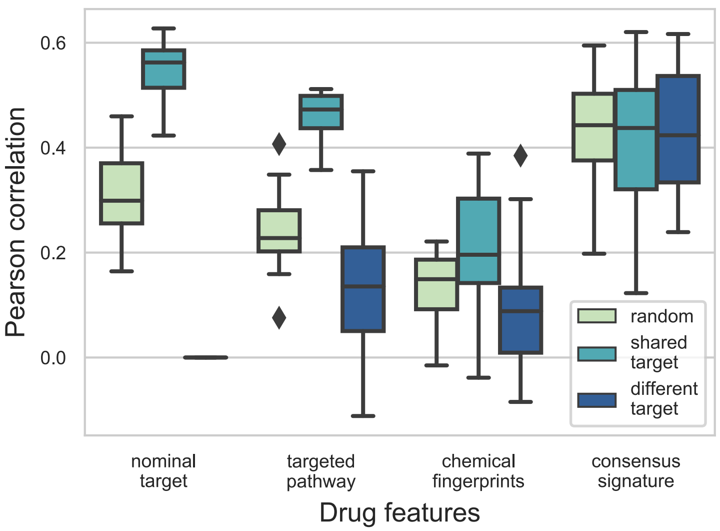

Supplement: gkz805_Supplemental_File [file gkz805_supplemental_file.pdf]
